# HDAC6 inhibitors sensitize non-mesenchymal triple-negative breast cancer cells to cysteine deprivation

**DOI:** 10.1038/s41598-021-90527-6

**Published:** 2021-05-26

**Authors:** Tahiyat Alothaim, Morgan Charbonneau, Xiaohu Tang

**Affiliations:** Department of Biological Sciences, Michigan Technological University, Houghton, MI 49931 USA

**Keywords:** Cancer, Drug discovery

## Abstract

Triple-negative breast cancer (TNBC) is a highly malignant type of breast cancer and lacks effective therapy. Targeting cysteine-dependence is an emerging strategy to treat the mesenchymal TNBC. However, many TNBC cells are non-mesenchymal and unresponsive to cysteine deprivation. To overcome such resistance, three selective HDAC6 inhibitors (Tubacin, CAY10603, and Tubastatin A), identified by epigenetic compound library screening, can synergize with cysteine deprivation to induce cell death in the non-mesenchymal TNBC. Despite the efficacy of HDAC6 inhibitor, knockout of HDAC6 did not mimic the synthetic lethality induced by its inhibitors, indicating that HDAC6 is not the actual target of HDAC6 inhibitor in this context. Instead, transcriptomic profiling showed that tubacin triggers an extensive gene transcriptional program in combination with erastin, a cysteine transport blocker. Notably, the zinc-related gene response along with an increase of labile zinc was induced in cells by the combination treatment. The disturbance of zinc homeostasis was driven by PKCγ activation, which revealed that the PKCγ signaling pathway is required for HDAC6 inhibitor-mediated synthetic lethality. Overall, our study identifies a novel function of HDAC6 inhibitors that function as potent sensitizers of cysteine deprivation and are capable of abolishing cysteine-independence in non-mesenchymal TNBC.

## Introduction

Triple-negative breast cancer (TNBC) accounts for 15–20% of overall breast cancer cases and exhibits earlier age of onset, high metastasis, and aggressiveness with poor clinical outcomes shown by higher relapse and lower survival rates than other types of breast cancer^1–3^. TNBC contributes to the majority of mortalities of breast cancer patients^4–6^. Treatments of TNBC patients are still limited to surgery, chemotherapy, or radiation since the absence of cell surface receptors makes targeted hormonal therapies impossible. About 50% of TNBC patients respond to conventional therapies, but the efficiency of treatments is further limited by tumor metastasis and drug resistance^7–11^. Therefore, there is an urgent need to develop novel targeted therapies to improve TNBC outcomes and reduce patient mortality.

With omics technologies, scientists have gained a deeper understanding of the molecular complexity of cancer^1,12,13^. Metabolic deregulation is one of the emergent hallmarks in many cancers^14,15^. Cellular metabolic rewiring often occurs with oncogenic alterations and microenvironmental adaptations to meet the demands of tumor cell survival, proliferation, and invasion^16–18^. Therefore, targeting metabolic vulnerabilities is a promising targeted strategy to treat these cancers. One classical example is that tumor cells acquire glycolysis (the Warburg effect) even under sufficient supply of oxygen. These tumors become addicted to glucose and are sensitive to inhibition of glycolysis by glucose analogues^19,20^. Similarly, alterations of amino acid metabolism are often observed in many cancers associated with genetic aberrations. For example, glutaminolysis is activated in *MYC* overexpressed tumors to feed the demands of lipids for fast proliferation, and thus *MYC*-induced tumors typically need extra glutamine^21,22^.

Cysteine dependence/addiction is a common metabolic vulnerability in many types of cancer^23–26^. Cysteine in cells, mostly derived from extracellular disulfide cystine, is involved in cellular glutathione (GSH) synthesis that removes cytotoxic reactive oxygen and nitrogen species (ROS and RNS) via the action of glutathione peroxidases (GPXs)^27^. Cysteine deprivation or inhibition of cystine/glutamate antiporter (the system xC-) by erastin or sulfasalazine can limit the GSH synthesis, subsequently accumulate lipid peroxidative stress in cells and ultimately induce ferroptosis in cysteine-dependent tumor cells^23^. Alternativity, application of “cysteinase” in vivo has been shown to be a safe approach to suppress tumor growth in mice with effective efficacy^28,29^. Therefore, targeting cysteine-dependence could be an effective targeted cancer therapy, especially because limiting a single amino acid is a relatively feasible method for in vivo application^30,31^. Cysteine dependence is a striking feature of mesenchymal TNBC^26^. However, many TNBC and luminal breast tumor cells are cysteine-independent and not effectively responsive to depletion of cysteine. Little is known about the mechanisms that dictate the demand of cysteine in TNBC.

Epigenetic alterations are important factors that regulate cancer development and contribute therapeutic drug resistance in cancer^32,33^. In breast cancer, distinct DNA methylation patterns are observed in luminal and basal cancer subtypes when analyzing the omics of 802 breast tumor cases. High DNA methylation and histone methylation were found in aggressive metastatic breast cancer^34–37^. Such epigenetic differences probably configure various subtypes of breast cancer and dictate cysteine dependence. Therefore, manipulation of epigenetic states in cysteine-independent cancer cells could change cysteine-dependence and overcome drug resistance.

By epigenetic compound library screening, we identified HDAC6 inhibitors as potent sensitizers that confer cysteine-dependence in non-mesenchymal TNBC. We showed that HDAC6 inhibitors induce the synthetic lethality of cysteine deprivation. This event occurs via an HDAC6-independent manner and requires activation of PKCγ signaling pathway. Our study suggested that HDAC6 inhibitors can be used as therapeutic adjuvants of cysteine deprivation to treat various non-mesenchymal breast cancers.

## Results

### HDAC6 inhibitors sensitize non-mesenchymal TNBC to cysteine deprivation

**Figure 1:**
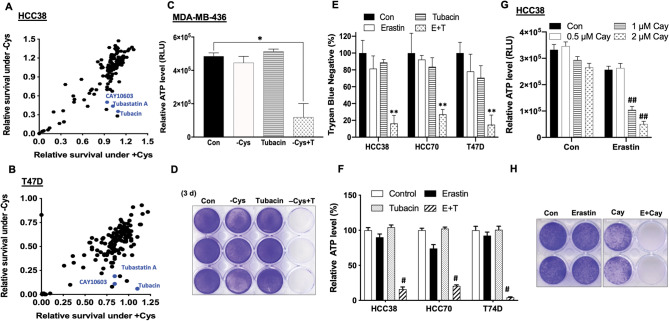
Epigenetic compound library screening identifies HDAC6 inhibitors to promote cell death of cystine deprivation. **(A,B)** Relative cell survival of TNBC HCC38 and luminal T47D cells treated with epigenetic compounds under cystine-depleted (-Cys) and cystine-replete (+ Cys) conditions were determined by CellTiter-Glo assay at 72 h. **(C,D)** Cell viability of TNBC MDA-MB-436 cells was measured by ATP level (**C**; n = 3; *p < 0.01) or stained by crystal violet (**D**) under either cystine-replete (Con), cystine-depleted (-Cys), 5 μM tubacin (T), or 5 μM tubacin under cystine-depleted (-Cys + T) treatments for 72 h. **(E,F)** Survival rate of HCC38, HCC70, and T47D cells in response to either control (Con), 5 μM erastin, 5 μM tubacin, or combination of erastin and tubacin (E + T) were determined by trypan blue counting (**E**; **p < 0.001) or CellTiter-Glo assay (**F**; n = 3; ^**#**^p < 0.001). **(G)** Cell viability of HCC38 cells was measured by CellTiter-Glo assay under either control (Con), 5 μM erastin, various concentrations of Cay10603 (Cay) 0.5, 1, and 2 μM, or erastin plus Cay (E + Cay) treatments for 48 h (n = 3; ^**##**^p < 0.005). **(H)** Relative cell survival of HCC38 was assessed by crystal violet staining after treatment with Con, 5 μM erastin, 2 μM Cay, or E + Cay for 48 h.

Cysteine-dependence is a novel feature in mesenchymal TNBC cells. However, we found that ~ 50% of TNBCs are non-mesenchymal, and enriched expression of epithelial genes (Fig. S1A–C). These non-mesenchymal TNBC cells, such as HCC70 and HCC38, were cysteine-independent and resistant to erastin, a blocker of cysteine transport (Fig. S1D), distinct from mesenchymal HBL100 and MDA-MB-231 cells. To overcome such resistances in non-mesenchymal tumor cells and identify potential sensitizers, three inhibitors of the histone deacetylase 6 (HDAC6)—Tubacin, CAY10603, and Tubastatin A were identified by the epigenetic compound library screening, which dramatically induce synthetic-lethal death under the cysteine-depleted condition (Fig. 1A,B). We further confirmed that tubacin induced synthetic-lethal cell death in non-mesenchymal MDA-MB-436 and HCC70 cells when cotreated with either cysteine deprivation or erastin; Either tubucin or erastin alone had no significant cytotoxic effect (Fig. 1C,D and Fig. S2A,B). The effectiveness of combined application of tubacin plus erastin was further observed in other tumor subtypes, including luminal and HER-2 positive tumor cells (Fig. 1E,F and Fig. S2C). Similarly, CAY10603 significantly promoted the lethal effects of erastin in non-mesenchymal TNBC as well (Fig. 1G,H and Fig. S2D). Colony formation assay further confirmed that tubacin plus erastin siginificantly suppressed long-term tumor cell growth in comparison with individual drug treatmnet (Fig. S2E). However, the synergistic effect of tubacin and erastin was not evident in MCF10A, a non-cancerous cells (Fig. S2F). Taken together, our epigenetic drug screening identified HDAC6 inhibitors as potent sensitizers capable of promoting synthetic lethality of cysteine depletion in non-mesenchymal tumor cells.

### HDAC6 inhibitors synergize with erastin to induce a mixed cell death

**Figure 2:**
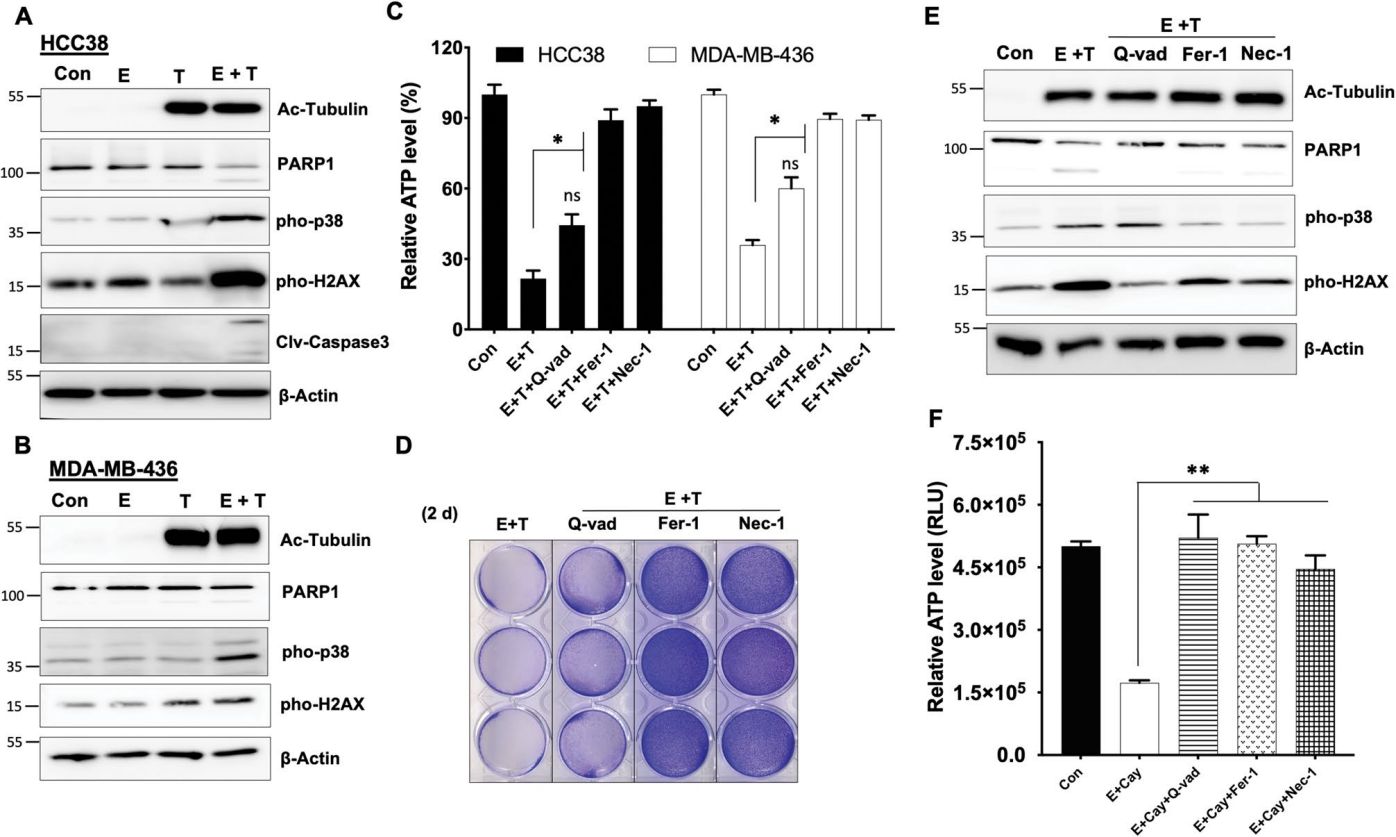
HDAC6 inhibitors synergize with erastin to induce a mixed type of cell death. **(A,B)** Immunoblot analysis of acetylated-tubulin, PARP1, cleaved Caspase-3, phosphorylated p38, and H2AX protein expression in HCC38 cells (**A**; 24 h) and in MDA-MB-436 cells (**B**; 36 h) under either control (Con), 5 μM erastin (E), 5 μM tubacin (T), or combination of erastin and tubacin (E + T) treatments; β-Actin serves as a protein normalization control. **(C, D)** Relative cell survival of HCC38 (48 h) and MDA-MB-436 (72 h) were measured by CellTiter-Glo assay (**C;** n = 3; *p < 0.001) or assessed by crystal violet staining in HCC38 (**D**) under either Con, combination of E + T, or E + T with different cell death inhibitors Q-Vad (10 μM), Fer-1(10 μM), Nec-1 (20 μM) treatments for 48 h. (**E**) Immunoblot analysis of indicated protein expression in HCC38 cells treated as (**C**) for 24 h. **(F)** Relative cell survival of HCC38 cells under either control (Con), combination of erastin and 2 μM Cay (E + Cay), or E + Cay with following death inhibitors Q-Vad 10 μM, Fer-1 (10 μM; **p < 0.005), Nec-1 (20 μM; **p < 0.005) treatments for 48 h.

To characterize the death pathway induced by the combination application of erastin and HDAC6 inhibitor, we examined various death and signaling markers, and evaluated the protective effect of different types of cell death inhibitors. Erastin plus tubacin treatment caused significant activation of p38 death signaling, increases of DNA double-strand breaks as indicated by phosphorylation of H2AX, and partial cleavages of PARP1 and caspase-3 (Fig. 2A,B), while either erastin or tubacin individually failed to activate such death signaling and markers. As the substrate of HDAC6, α-tubulin was strongly acetylated by tubacin. Both necroptosis inhibitor Necrostatin-1 and ferroptosis inhibitor Ferrostatin-1 fully rescued cells from death induced by erastin plus tubacin, while the pan-caspase inhibitor Q-Vad had partial protective effects (Fig. 2C–E). Similarly, the lethal effects promoted by CAY10603 were prevented by different types of cell death inhibitors (Fig. 2F). This data suggested that HDAC6 inhibitors synergize with erastin to activate a mixed cell death program in non-mesenchymal TNBC.

### HDAC6 is not required for the tubacin-mediated synthetic lethality.

**Figure 3:**
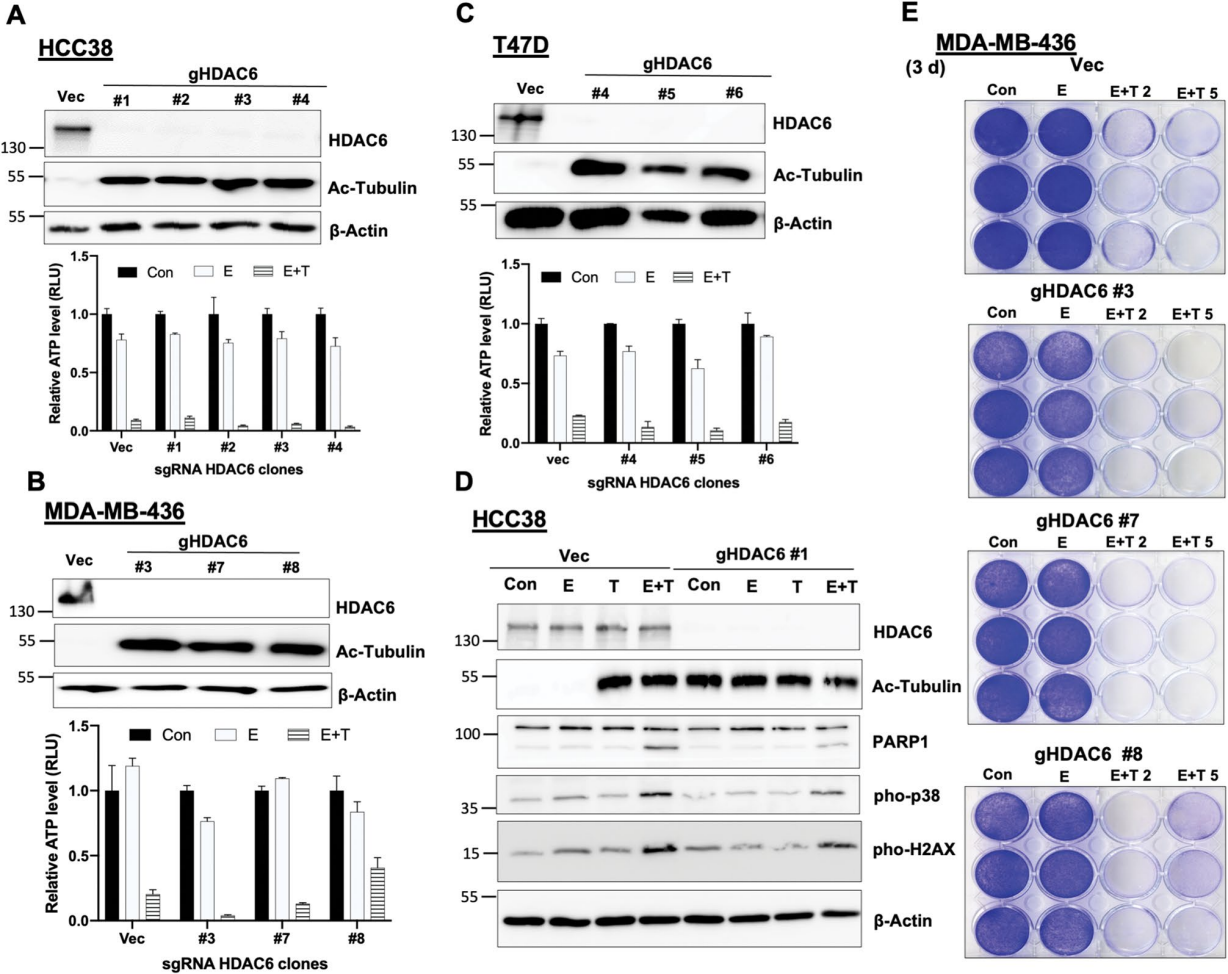
Knockout of HDAC6 fails to mimic tubacin to induce synergistic cell death. **(A–C)** Immunoblotting analysis (Upper panel) of HDAC6 and acetylated tubulin in the empty vector (Vec) cells and sgRNA HDAC6 (gHDAC6) clones of HCC38 (**A**), MDA-MB-436 (**B**), and T47D (**C**) cells. Relative cell survival (Lower panel) was measured by the ATP level in indicated cells under either control (Con), 5 μM erastin, or combination of erastin and 5 μM tubacin (E + T) treatment for 72 h (n = 3). **(D)** Western blot analysis of indicted protein expression in HCC38 Vec or gHDAC6 HCC38 cells (Clone #1) under either control (Con), 5 μM erastin (E), 5 μM tubacin (T), or combination of erastin and tubacin (E + T) treatments for 24 h. **(E)** Cell viability was assessed by crystal violet staining in MDA-MB-436 Vec or gHDAC6 clones under either control (Con), erastin (E), or erastin plus different doses of tubacin (E + T) treatments for 72 h.

Next, we examined whether repression of HDAC6 expression mimics the lethal-promoting effect of tubacin. Unexpectedly, silencing of HDAC6 gene expression by different shRNAs did not promote cell death in the presence of erastin, although the protein level of HDAC6 was reduced and the acetylation level of tubulin was significantly increased (Fig. S3A–C). We hypothesized that shRNA might be unable to sufficiently suppress endogenous HDAC6 to mimic the potency of tubacin. To that end, we employed CRISPR/Cas9 gene editing to knockout HDAC6 in TNBC HCC38 and MDA-MB-436 cells, and luminal T47D cells. Independent HDAC6-null cell clones were isolated based on deletion of HDAC6 protein expression and acetylation of tubulin (Fig. 3A–C upper panel). Surprisingly, knockout of HDAC6 also did not promote the synthetic-lethal response with erastin as shown by no significant ATP decrease and cell loss in HDAC6-null cell clones (Fig. 3A–C lower panel and 3E). However, erastin plus tubacin together induced similar cell death (Fig. 3A–C lower panel and 3E) and death markers (Fig. 3D) in either HDAC6-null or HDAC6 intact cells, despite the HDAC6 status. Taken together, our data provided evidence that the lethal-promoting effect of HDAC6 inhibitors is independent of endogenous HDAC6, which revealed the possibility of a new off-target molecule. The sphingolipid biosynthesis was previously identified as a so-called off-target of tubacin^38^. However, myriocin, an inhibitor of sphingolipid synthesis, failed to promote cell death in the presence of erastin in non-mesenchymal tumor cells (Fig. S3D,E).

### Tubacin synergizes with erastin to activate a lethal gene transcriptional program

**Figure 4:**
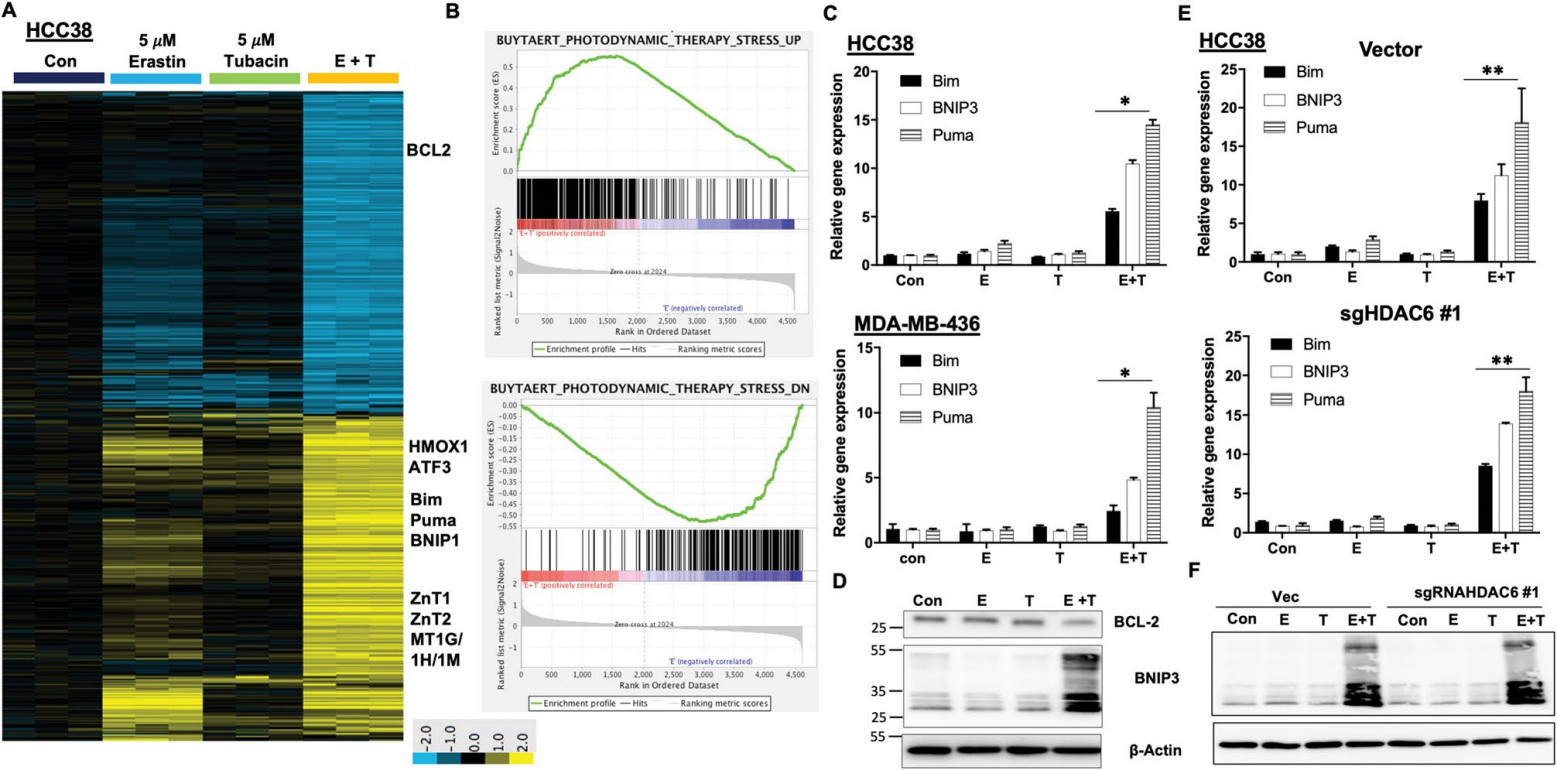
Tubacin synergizes with erastin to induce an extensive gene transcriptional response. **(A)** Heatmap cluster view of transcriptional profiling in HCC38 cells under treatments of either control (Con), 5 μM erastin (E), 5 μM tubacin (T), or combination of erastin and tubacin (E + T) for 24 h. **(B)** Gene enrichment of GSEA analysis in the gene expression profile induced by E + T. **(C)** RT-qPCR analysis of apoptotic gene expression in HCC38 and MDA-MB-436 (n = 3; *p < 0.001) cells treated as (**A**). **(D)** Immunoblot analysis of BCL-2 and BNIP3 protein expression in HCC38 cells. **(E)** RT-qPCR analysis of apoptotic gene expression (^#^p < 0.0001) in HCC38 Vector and gHDAC6 #1 cells treated as (**A**). **(F)** Immunoblot analysis of BNIP3 protein expression in HCC38 Vec and gHDAC6 #1 cells.

Microarray transcriptional profiling analysis was employed to identify the molecular mechanism of tubacin. By the supervised cluster analysis, either erastin or tubucin individually induced mild or few changes in gene expression. However, tubacin plus erastin dramatically induced or repressed expression of large groups of genes (Fig. 4A), such as apoptotic genes *(Bim*, *BNIP3*, and *Puma*) and genes involved in endoplasmic reticulum and oxidative stress (*ATF3*, *CHOP*, and *HOMX1*). GSEA revealed that the gene responses activated by erastin plus tubacin are similar to gene changes induced by photodynamic therapy (PDT) (Fig. 4B), which is a well-established cancer therapy to cause tumor ablation^39^. RT-qPCR and immunoblotting data confirmed that apoptotic genes were strongly induced by the combination treatment, but not by either erastin or tubacin alone (Fig. 4C,D). Gene changes induced by erastin plus tubacin were similar in HDAC6-null cells in comparison with HDAC6 wild type cells (Fig. 4E,F). Taken together, the lethal gene response of tubacin plus erastin reiterated the potential of tubacin to confer cysteine-dependence in non-mesenchymal TNBC via an HDAC6-independent manner.

### Tubacin synergizes with erastin to induce cellular zinc response

**Figure 5:**
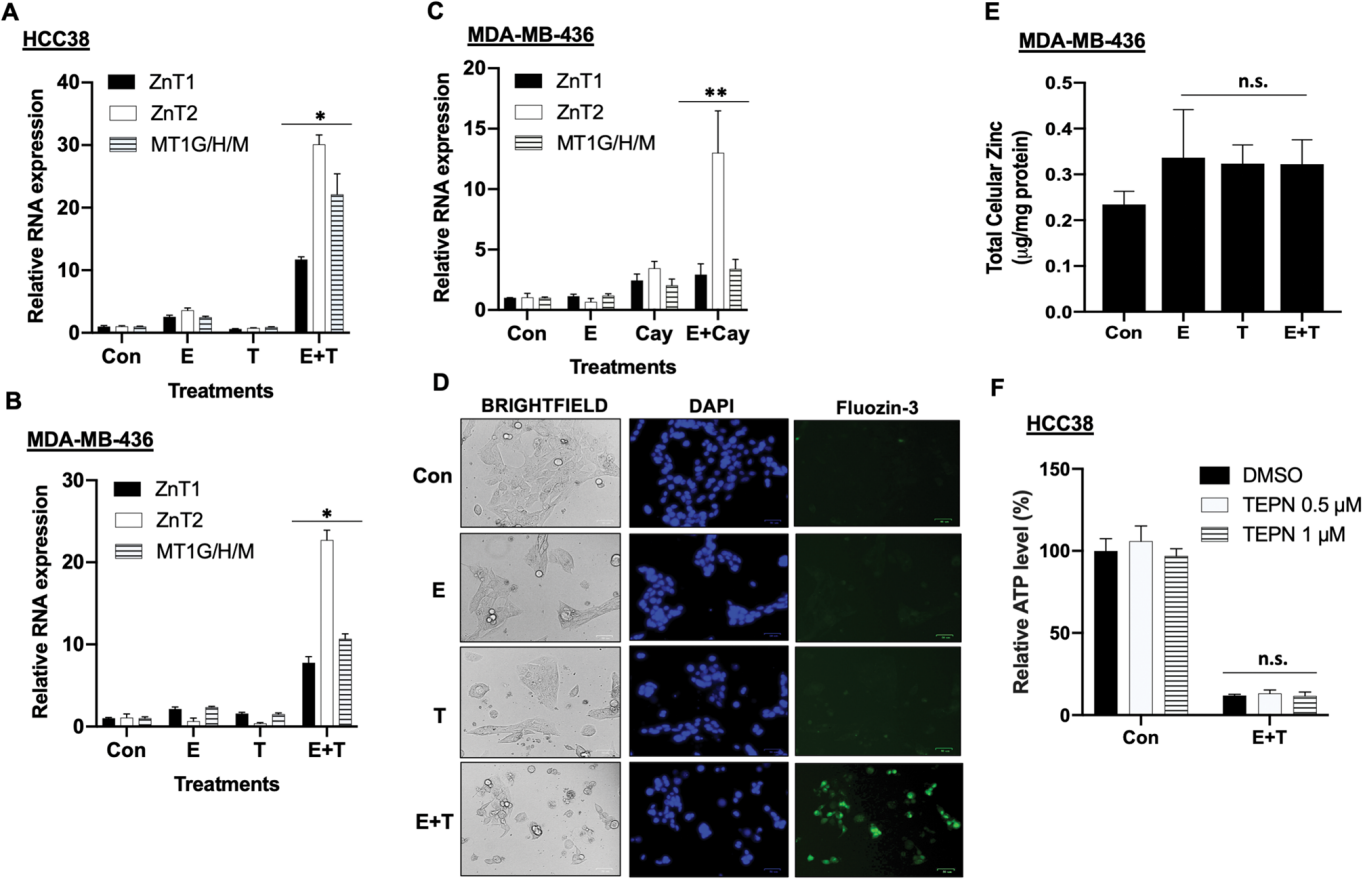
The zinc-related gene response is triggered by erastin plus tubacin. **(A,B)** RT-qPCR analysis of zinc-related gene expression (n = 3; *p < 0.005) in HCC38 cells (**A**; 24 h) and MDA-MB-436 (**B**; 36 h) under treatments of either control (Con), 5 μM erastin (E), 5 μM tubacin (T), or erastin plus tubacin (E + T). **(C)** RT-qPCR analysis of zinc-related gene expression (n = 3; **p < 0.005) in MDA-MB-436 (36 h) under treatments of either control (Con), 5 μM erastin (E), 5 μM Cay10603 (Cay), or erastin plus Cay10603 (E + Cay). **(D)** Live cell imaging of HCC38 cells that were treated as (**A**) for 18 h and stained by Fluozin-3 and DAPI (Hoechst 33342). The size of the scale bar is 100 μm. **(E)** Total cellular zinc was measured by ICP-AES analysis (n = 3; n.s., not significant) in MDA-MB-436 cells under either control (Con), 5 μM erastin (E), 5 μM tubacin (T), or erastin plus tubacin (E + T) treatments for 24 h. **(F)** Relative cell viability of HCC38 cells under either control or E + T with different concentrations of TPEN for 48 h (n = 3; n.s., not significant).

Ferroptosis is an iron-dependent cell death that is able to be induced by erastin. In our gene expression profiling, gene changes involved in iron metabolism were not observed. Interestingly, the genes involved in zinc transport, ZnT1 and ZnT2, as well as the genes related to zinc storage, metallothioneins MT1G/1H/1 M, were highly induced by erastin plus tubacin (Fig. 4A). RT-qPCR confirmed that these zinc-related genes were strongly induced by erastin plus tubacin, but not by either erastin or tubacin alone (Fig. 5A,B). Similarly, CAY10603 in combination with erastin strongly activated the zinc-related gene response (Fig. 5C). Zinc is known to cause ROS production and induce a mixed type of cell death, including apoptosis and necrosis^40,41^. Therefore, we examined whether the level of zinc is altered by erastin plus tubacin. Indeed, FluoZin™-3, a Zn^2+^-selective indicator, detected that the level of labile zinc was highly increased in cells by erastin and tubacin, but not significantly by either erastin or tubacin alone (Fig. 5D and Fig. S4A). Labile zinc mostly accumulated in the nucleus, as it co-localized with nuclear DNA. The total cellular amount of zinc measured by ICP-OES, including both labile and bound zinc, was not significantly altered under any treatments (Fig. 5E), indicating that the labile zinc originated from zinc cellular proteins or compartments; it was not imported from culture media. However, chelation of labile zinc with TPEN^42^ did not protect cells from cell death induced by erastin plus tubacin (Fig. 5F and Fig. S4B), indicating that labile zinc is not the causative agent of cell death.

### Inhibition of PKC suppresses the synthetic lethality of tubacin

**Figure 6:**
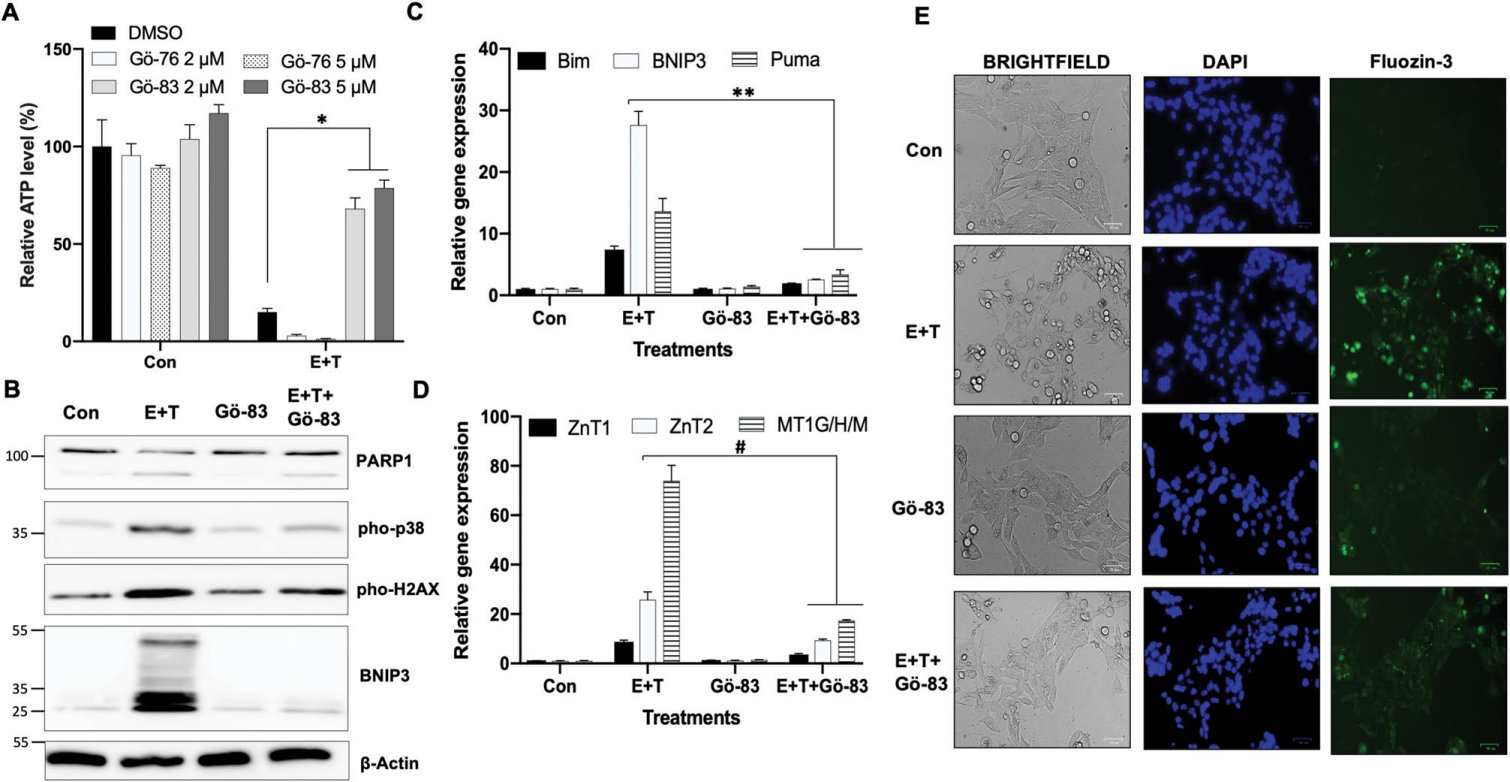
Inhibition of PKC suppresses increase of labile zinc and cell death. **(A)** Relative cell survival of HCC38 cells under either control or 5 μM erastin and 5 μM tubacin (E + T) treatments with or without PKC inhibitor Gö 6976 or Gö 6983 for 72 h (n = 3; *p < 0.005). **(B)** Western blot analysis in HCC38 cells that were treated as (**A**) for 24 h. **(C,D)** RT-qPCR expression analysis of apoptotic genes (**C**; **p < 0.001) and zinc-related genes (**D**; ^#^p < 0.0001). **(E)** Living cell imaging of HCC38 cells that were treated as (**A**) for 18 h and stained by Fluozin-3 and DAPI (Hoechst 33342). The size of the scale bar is 50 μm.

Considering that zinc functions as a structural or modulatory component of many regulatory and signaling proteins^43,44^, the increase of labile zinc in cells is probably an indication of cellular protein function or signaling alterations. Particularly, zinc release from protein kinase C (PKC) is a common event during PKC activation by reactive oxygen species (ROS) during cell death^45,46^. Therefore, we examined the role of PKC in the synthetic lethality of tubacin. We found that Gö 6983, a PKC inhibitor with a broad inhibitory spectrum^47^, rescued cells from cell death induced by erastin and tubacin, while Gö 6976, a selective inhibitor of PKC $\alpha/\beta$, had no protective role (Fig. 6A and Fig. S5A). Immunoblotting and RT-qPCR analysis confirmed that Gö 6983 abolished phosphorylation of PKC substrates and the induction of cell death genes induced by erastin plus tubacin (Fig. 6B,C and Fig. S5B). Moreover, Gö 6983 significantly suppressed the zinc-related gene response and decreased labile zinc in cells (Fig. 6D,E and Fig. S5C). These results suggested that activation of PKC, but not PKC $\alpha/\beta$, is required for the tubacin-mediated cell death.

### PKCγ is required for the tubacin-mediated synthetic lethality

**Figure 7:**
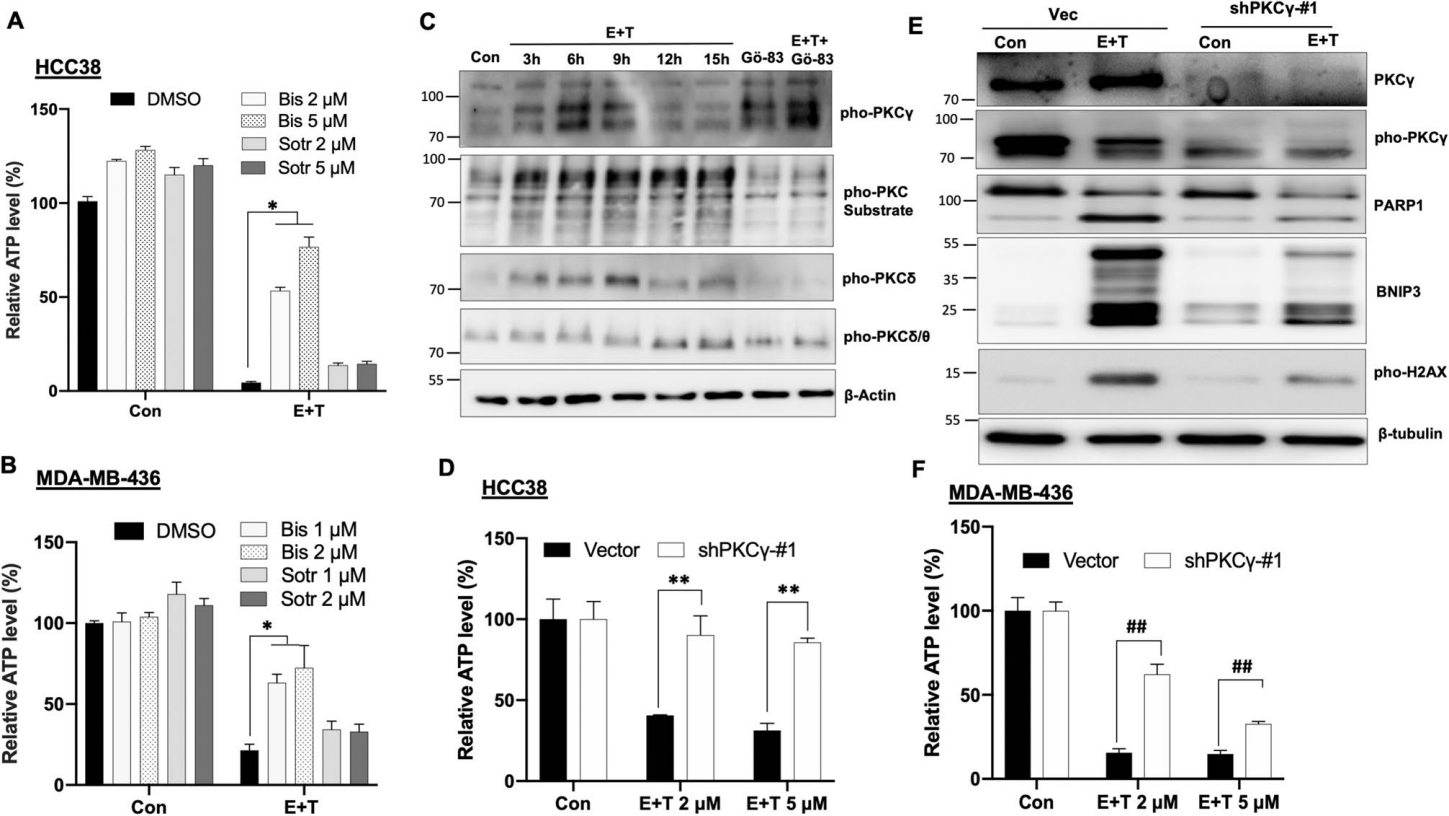
PKCγ is required for the tubacin-mediated cell death. **(A,B)** Relative cell survival of HCC38 (**A;** n = 3; *p < 0.001) and MDA-MB-436 (**B;** n = 3; *p < 0.005) under either control (Con) or 5 μM erastin plus 5 μM tubacin (E + T) treatments with or without PKC inhibitors bisindolylmaleimideI (Bis) or sotrastaurin (Sotr) for 72 h. **(C)** Immunoblotting protein analysis in MDA-MB-436 cells under either Con or E + T treatments with or without Gö 6983 for indicated times. **(D)** Cell viability of HCC38 Vec and shPKCγ-#1cells under either Con, or erastin (5 μM) plus different doses of tubacin for 48 h (n = 4; **p < 0.01). **(E)** Immunoblotting protein analysis in HCC38 Vec and shPKCγ-#1cells under either Con or erastin plus tubacin for 24 h. **(F)** Cell viability of MDA-MB-436 Vec and shPKCγ-#1 cells under either Con or erastin (5 μM) with different doses of tubacin for 72 h (n = 4; ^**##**^p < 0.01).

To examine which member of PKC family is required for the synthetic lethality of tubacin, two additional PKC inhibitors, bisindolylmaleimide I and sotrastaurin, with slightly different inhibitory spectrums were used. Similar to Gö 6983, bisindolylmaleimide I strongly protected cells from cell death induced by erastin plus tubacin, but sotrastaurin did not (Fig. 7A,B). Based on the protective role and inhibitory spectrums of various PKC inhibitors, PKCγ stood out as the candidate kinase since it is inhibited by Gö 6983 and bisindolylmaleimide I, but not by Gö 6976 and sotrastaurin. Additionally, PKCγ was transiently phosphorylated in the early phase and associated with the phosphorylation of PKC substrates during the erastin plus tubacin treatment. On the other hand, the phosphorylation of PKC δ/θ occurred in the later phase (Fig. 7C). Knockdown of PKCγ by shPKCγ significantly alleviated cell death induced by erastin plus tubacin in non-mesenchymal TNBC cells (Fig. 7D,F and Fig. S6A). Consistent with the protective role of PKCγ, its knockdown suppressed induction of cell death markers (PARP1, pho-H2AX, and BNIP3) (Fig. 7E and Fig. S6A,B). Taken together, activation of PKCγ is required for the synthetic lethality of tubacin.

## Discussion

TNBC is the most challenging subtype of breast cancer and overall its survival rate is low^48^. Developing novel and precise targeted therapies is urgently needed. Targeting cancer metabolism emerges as a promising strategy to treat some cancers with metabolic deregulations. Particularly, cysteine depletion can eradicate a subset of TNBC with highly mesenchymal states (EMT)^49^. However, many TNBCs with epithelial features, as well as luminal breast cancers are cysteine-independent and unresponsive to cysteine deprivation. Epigenetic alterations can change tumor identity, metabolism, heterogeneity, and result in drug resistances in cancer^35,37^. Many epigenetic activators or inhibitors are employed as therapeutic adjuvants to enhance chemotherapy efficacy and overcome drug resistance^32,33^. In line with these ideas, we identified that HDAC6 inhibitors sensitize non-mesenchymal TNBC cells and some luminal cancer cells to cysteine deprivation.

HDAC6 is a unique member of the HDAC family that can regulate cell proliferation, metastasis, and invasion in tumors, and can also drive tumor progression and confer drug resistance in some cancers^50–53^. Although HDAC6 is likely a reasonable molecular target in our context, we have shown that knockout of HDAC6 protein expression does not mimic the effects of HDAC6 inhibitors; endogenous HDAC6 removal does not synergize with erastin to induce cell death in the non-mesenchymal TNBC. These suggest that the synergistic effect of HDAC6 inhibitors occurs via a new cellular molecule or pathway that is entirely independent of HDAC6. We aim in future studies to identify such genes or pathways as they can be valuable direct targets to be exploited in combination with cysteine deprivation for cancer treatment.

Although the intrinsic target of HDAC6 inhibitors in this study remains unknown, we found that tubacin plus erastin triggered many stress and apoptotic gene responses, one of which mimics the gene response of photodynamic therapy (PDT)^39^. The synthetic lethality of tubacin exhibited as a mixed type of cell death, including apoptosis, necroptosis, and ferroptosis. Ferroptosis is an iron-dependent cell death driven by lipid peroxidation with necrotic features^54–56^. Our transcriptional profiling data didn’t show any iron-related gene response; instead, the expression of several zinc-related genes and the increase of labile zinc was prominently induced by erastin plus tubacin.

Zinc is a trace but essential metal micronutrient and is integral to many enzymes and regulatory proteins, and functions as a signaling messenger in cells^57,58^. Although cellular zinc homeostasis is disturbed by tubacin plus erastin, we observed that the total amount of zinc, including labile and bound forms, was not significantly changed in treated cells; also, chelation of labile zinc didn’t protect cells from death. These suggested that labile zinc is not the direct causative agent for cell death, instead, it is an indicator of intracellular signaling perturbations. It’s known that reactive oxygen species (ROS) can cause zinc releasing from oxidized metallothioneins and zinc-fingers of abnormal proteins^59^. For example, activation of PKC $\alpha /\beta$ by ROS can release zinc directly from PKC $\alpha /\beta$ zinc fingers into the cytoplasm^45,46^. Indeed, inhibition of PKC abolished the release of labile zinc and cell death in our study. Specifically, activation of PKCγ is required for the synthetic lethality of tubacin. As one of the conventional PKC isozymes, PKCγ is mainly present in the brain and has rarely been explored in studies on cancer^60^. Recent studies showed that activation of PKCγ increases the migratory capacity of colon cancer^61,62^. Our study identified a new role of PKCγ in breast cancer, whereby the PKCγ signaling mediates the synthetic lethality of tubacin in non-mesenchymal TNBC cells.

Taken together, many TNBC cells are non-mesenchymal and cysteine-independent. HDAC6 inhibitors identified in our study, particularly tubacin, can overcome the resistance of cysteine deprivation in non-mesenchymal TNBC and promote the synthetic lethality of cysteine deprivation. These inhibitors execute their lethal effects independent of their canonical target, the HDAC6 protein. Instead, HDAC6 inhibitors, in synergism with erastin, trigger an extensive gene transcriptional program to induce cell death via the PKCγ signaling. HDAC6 inhibitors will be immensely valuable as adjuvants in the application of targeted cysteine-dependence therapy to treat various types of breast cancer.

## Methods

### Cell culture and reagents

All breast tumor cells and 293 T cells were purchased from ATCC and maintained as per standard protocols in an incubator with 95% humidity and 5% CO_2_ at 37 °C. Cells were cultured in DMEM with 10% heat-inactivated fetal bovine serum (FBS) and 1% penicillin–streptomycin. Cysteine deficient medium was prepared according to the previous report^63^. MCF10A cells were maintained as per a standard protocol of ATCC and cultured in MEGM supplemented with MEGM bullet kit. Erastin, selective HDAC6 inhibitors tubacin, tubastatin A, and CAY10603, Myriocin, and the metal chelator *N*, *N*, *N′*, *N′*-tetrakis (2-pyridylmethyl) ethylenediamine (TEPN) were obtained from Cayman Chemicals (Ann Arbor, Michigan, US); Z-VAD-FMK, necrostatin-1, and ferrostatin-1 were purchased from Calbiochem Research Biochemicals (Sigma). The molecular probe FluoZin-3 was purchased from Thermo Fisher Scientific. All antibodies used in this study were listed in supplementary table 1.

### Epigenetic compound library screening

The epigenetic compound library screening was performed in TNBC HCC38 and luminal T47D cancer cells. 140 inhibitors of epigenetic regulators were included in the compound library (Cayman chemical). Briefly, cells were seeded in two sets of 96-well plates under either cysteine-rich or cysteine depleted conditions. Each epigenetic compound at the final concentration of 2 μM was applied to these cells. Cell viability was determined at 72 h using the CellTiter-Glo assay kit (Promega).

### Lentiviral cell infection

Viral particles were generated in 293T cells by transfecting lentiviral packaging plasmids using Lipofectamine 3000 (ThermoFisher Scientific) and collected from cell media after 48 h. The targeted cells were infected for 48 h by indicated viruses and further selected by either puromycin or blasticidin (Cayman chemical). The pLentiCRISPR-v2-HDAC6 was purchased from GenScript with the targeting sgRNA sequence 5’-CAGTGCTACAGTCTCGCAC. Independent HDAC6-null cell clones were isolated based on the complete deletion of HDAC6 protein expression and increased acetylation of tubulin. All shRNAs targeting *HDAC6* and *PKC*γ genes were purchased from Sigma. The pLKO1.0 plasmid was used as a control vector for infection.

### Gene expression profiling and geneset enrichment analysis (GSEA)

The gene expression profile in HCC38 cells treated with either control, erastin, tubacin, or erastin plus tubacin in triplicate for 24 h was analyzed by the GeneChip™ Human Gene 2.1 ST 24-Array Plate (ThermoFisher Scientific). The data were deposited in the GEO database (GSE154425). Probe intensities were normalized by RMA module. Gene expression changes in treated HCC38 cells were derived by zero-transformation (Δlog_2_) against those in the control condition. Probe sets that varied by twofold in at least 3 samples were selected for hierarchical clustering. The pathway enrichment was analyzed by Gene Set Enrichment Analysis (GSEA) module using the G2 annotated-genesets with default criteria of 1000 permutations.

### RNA extraction and real-time RT-PCR

RNA was extracted from cells by RNeasy kit (Invitrogen). Total RNA (2 µg) was reverse-transcribed to cDNA and the quantitative PCR was performed using SYBR Green PCR master mixture (Applied Biosystems). The relative difference in gene expression was normalized with the Actin B gene expression using the ΔΔCT method. All primers in this study were listed in supplementary table 2.

### Protein immunoblotting analysis

Proteins were extracted from cells using RIPA extraction and lysis buffer (Sigma) with the protease and phosphatase inhibitor cocktail (ThermoFisher Scientific). Protein concentrations were determined by BCA protein assay. Equal amounts of protein were loaded for immunoblot analysis. Signals were detected by the ECL plus Western blotting detection system (Amersham) and visualized by LAS-4000 lumino image analyzer.

### Cell viability and cytotoxicity

Cell viability was measured by either counting of trypan blue negative cells, relative ATP levels using CellTiter-Glo assay kit (Promega), or evaluation by crystal violet staining. Cell cytotoxicity was measured as protease release using CytoTox-Fluor cytotoxicity assay kit (Promega).

### Clonogenic assay

Cells were seeded at a density of 5 × 10^3^ cells per well in 12-well plates and incubated for two days. Then, the cells were treated with drugs for three days, and replaced with fresh culture medium for additional two or three weeks at 37 °C. Finally, colonies were stained with 1% crystal violet at room temperature.

### Microscopic imaging

To analyze intracellular zinc levels, 7 × 10^3^ cells were seeded in the 96-well plate under various treatments. At the end, cells were stained by a 2 μM FluoZin-3 molecular probe (ThermoFisher Scientific) at 37 °C for 30 min. Dialyzed FBS medium was used to ensure that the medium was zinc-free. Additionally, cells were also stained by 1 µM of 4′,6-diamidino-2-phenylindole (DAPI) for 5 min. Cells were then imaged by ZOE™ Fluorescent Cell Imager microscopy.

### Cellular zinc level measurement

Total cellular zinc, including labile and bound zinc, was determined by ICP-OES (CHORI Elemental Analysis Facility)^64^. Briefly, 3 × 10^6^ HCC38 cells under different treatments were collected and fully dissolved in 0.25 mL OmniTrace 70% HNO_3_ (EMD Chemicals) by microwave digestion. Samples were diluted to 5% HNO_3_ with OmniTrace water and analyzed with the use of a Vista Pro ICP-OES (Varian Vista Pro). Zinc was measured at the 213-nm wavelength with a detection range between 0.005 and 5 ppm. All associated reagents and plasticware were certified as trace metal-free or tested for trace metal contamination. Zinc concentrations were normalized by total protein mass. All samples were analyzed in triplicate.

### Statistical analyses

The significance of differences between groups was determined using a *t* test. Statistical analysis was performed using GraphPad Prism version 8.3.1, GraphPad Software, San Diego, California USA, www.graphpad.com. A *p*-value < 0.05 was considered statistically significant. Data were presented in figures as mean ± standard deviation (SD).

## Supplementary Information

Supplementary Information.

## Data Availability

The datasets generated in the current study are available in the Gene Expression Omnibus (GEO) database (https://www.ncbi.nlm.nih.gov/geo/).
