# Supplementary material for: HDAC6 inhibitors sensitize non-mesenchymal triple-negative breast cancer cells to cysteine deprivation
Source: Sci Rep. 2021 May 26;11:10956. doi: 10.1038/s41598-021-90527-6 (PMC8155140; doi:10.1038/s41598-021-90527-6)
Supplement: Supplementary file 1 — Supplementary Information. [file 41598_2021_90527_MOESM1_ESM.pdf]

HDAC6 inhibitors sensitize non-mesenchymal triple-negative breast cancer cells to cysteine deprivation

Tahiyat Alothaim[1], Morgan Charbonneau[1], Xiaohu Tang[1, 2]

[1] Department of Biological Sciences, Michigan Technological University, Houghton, Michigan, 49931

[2] Corresponding author: Xiaohu Tang;

E-mail: xiaohut@mtu.edu;

Telephone: 906-487-3068

Conflict of Interest: The authors have declared no conflict of interests for this study.

Running title: Tubacin synergizes with erastin to eradicate TNBC

Supplementary Figure Legends

Figure S1. Cysteine-dependence associates with epithelial-mesenchymal transition (EMT) in TNBC

(A) Heatmap cluster viewing of gene expression in epithelial and mesenchymal TNBCs (The data from GSE69017).

(B) Gene enrichment of luminal tumor associated genes in epithelial TNBCs by Gene sets enrich analysis (GSEA).

(C) RT-qPCR analysis of indicated genes in luminal tumor cells, mesenchymal and non-mesenchymal TNBCs.

(D) Cell cytotoxicity of TNBC cells (Mesenchymal: MDA-MB-231 and HBL100; Non-mesenchymal: HCC70 and HCC38;) was measured by relative protease releasing under either control or 5 μM erastin treatments for 20 hrs (n=3; *, p<0.001) or crystal violet staining (3 days).

Figure S2. Epigenetic compound library screening identifies HDAC6 inhibitors as sensitizers of cysteine depletion in the non-mesenchymal TNBC

(A) Relative cell survival was measured by relative ATP level in MDA-MB-436 cells under either control (Con), 5 μM erastin (E), 5 μM tubacin (T), or erastin plus tubacin (E+T) treatments for 72 hrs (n=3; *, p<0.001). The cell viability was also assessed by crystal violet staining.

(B) Cell viability of HCC70 was assessed by crystal violet staining after exposure to the control, 5 μM erastin, 5 μM tubacin, or E+T for 72 hrs.

(C) Cell viability was measured by relative ATP level in luminal MDA-MB-361 and SUM-52, and HER2 positive SKBR3 with the similar treatments to (**A**) for 72 hrs (n=3; $^{\#}$**,** p<0.05).

(D) Relative cell survival of MDA-MB-436 was measured by CellTiter-Glo assay or assessed by crystal violet staining under either control, 5 μM erastin, or in combination with Cay10603 (n=3; **, p<0.001) treatments for 72 hrs.

(E) Cologenic growth of T47D, MDA-MB-436, and HCC38 under either control (Con), 5 μM erastin (E), 5 μM tubacin (T), or erastin plus tubacin (E+T) treatments.

(F) Relative cell viability was measured by relative ATP level in MCF10A under either control (Con), 5 μM erastin (E), 5 μM tubacin (T), or erastin plus tubacin (E+T) treatments for 72 hrs (n=3). The cell viability was also assessed by crystal violet staining.

Figure S3. Knockdown of HDAC6 expression does not mimic tubacin-mediated synthetic-lethality

(A, B, C) Immunoblotting analysis (Upper panel) of HDAC6 and acetylated tubulin in **(A)** HCC38, **(B)** MDA-MB-436, and **(C)** luminal T47D infected either with the empty pLKO vector (Vec) and different shHDAC6 constructs; Relative cell survival (Lower panel) was measured by relative ATP level in indicated cells under either control (Con) or erastin treatments for 72 hrs (n=3).

(D, E) Cell viability was measured by relative ATP level in HCC38 (**D**; 48 hrs) and MDA-MB-436 (**E**; 72 hrs) cells under either control or erastin plus indicated concentrations of myriocin (Myr; 10 or 20 μM) treatments.

Figure S4. **Erastin plus tubacin increases cellular labile zinc**

(A) Living cell imaging of MDA-MB-436 cells that was stained by Fluozin-3 and DAPI (Hoechst 33342) under the similar treatments to (**A)** for 18 hrs. The size of scale bar is 50 μm (n=3).

(B) Cell survival was measured by relative ATP level in MDA-MB-436 cells under either control or 3 μM of zinc chelator (TEPN) in combination with erastin plus tubacin after 72 hrs (n=3).

Figure S5. PKC activation is required for the tubacin-mediated cell death and labile zinc increasing

(A) Relative cell survival was measured by the ATP level in MDA-MB-436 under either control or 5 μM erastin plus 5 μM tubacin (E+T) treatments with or without different doses of PKC inhibitor Gö 6976 or Gö 6983 for 72 hrs (n=3; *, $p<0.005$).

(B) Immunoblot analysis of phosphorylated PKC (Pho-PKC) substrates in HCC38 cells under the similar treatments to (**A**) for 22 hrs.

(C) Living cell imaging of HCC38 cells that was stained by Fluozin-3 and DAPI (Hoechst 33342) under either E+T, or E+T with 5 μM Gö 6983 treatments for 18 hrs. The scale bar represents 30 μm.

Figure S6. PKCγ is required for the tubacin-mediated synthetic lethality

(A) Immunoblot analysis of PKCγ and death markers (PARP1, pho-H2AX) in HCC38 pLKO vector (Vec) and shPKCγ-#2 under either control or 5 μM erastin plus 5 μM tubacin (E+T) treatments for 24 hrs. Relative cell survival in indicated cells was measured by relative ATP level after 72 hrs treatment (Lower panel; n=3; *, $p<0.005$).

(B) Immunoblot analysis of PKCγ and phosphorylated PKCγ and p38 (Lower panel) in MDA-MB-436 pLKO vector (Vec) and shPKCγ-#1 cells under the similar treatments to (**A**) for 48 hrs.

Additional supplemental figures are full western blots for Main & Supplemental Figures.

Figure S1

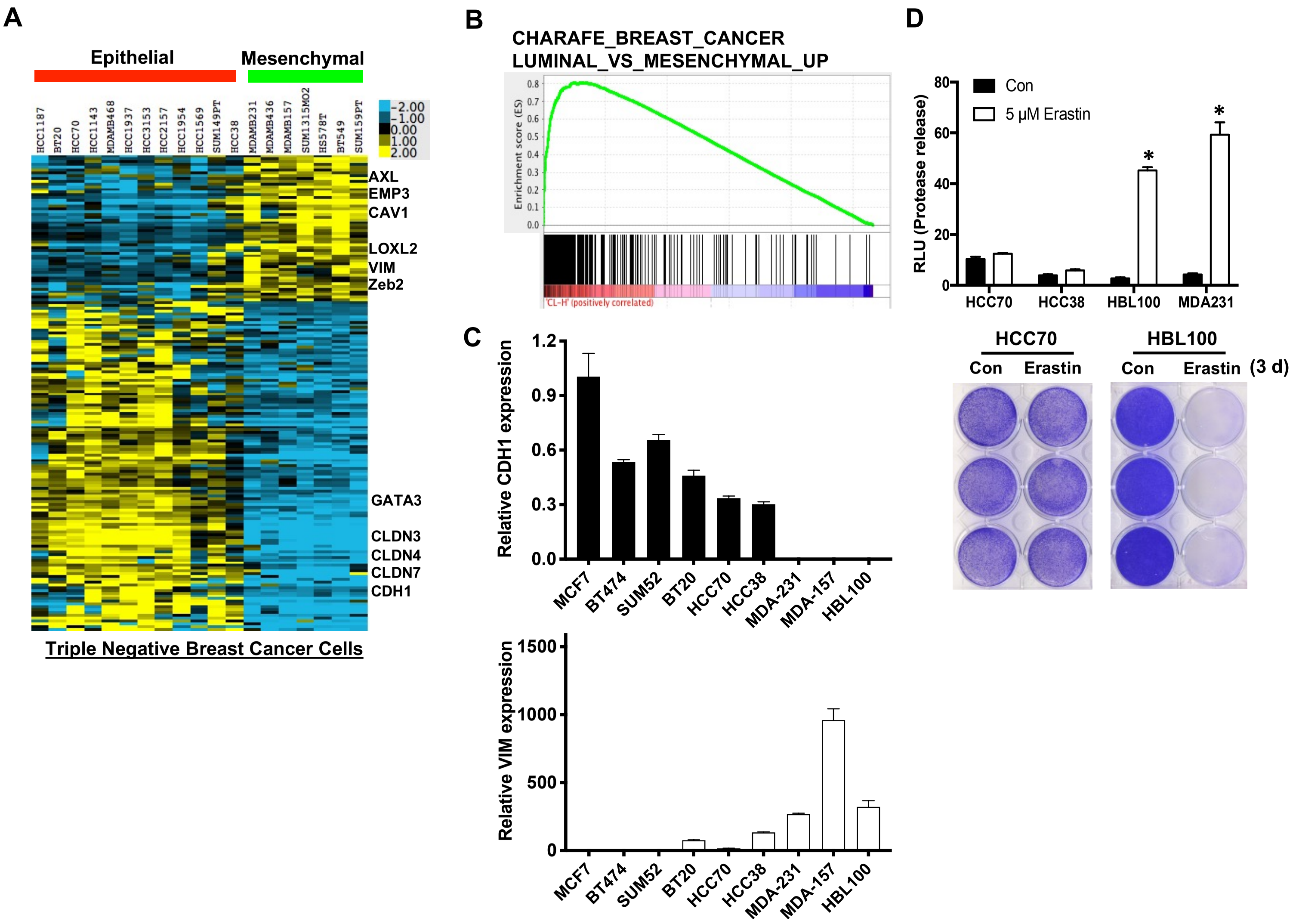

A
Epithelial
Mesenchymal
HCC1187
BT20
HCC70
HCC1143
MDAMB468
HCC1937
HCC3153
HCC2157
HCC1954
HCC1569
SUM149PT
HCC38
MDAMB231
MDAMB436
MDAMB157
SUM1315MO2
HS578T
BT549
SUM159PT
-2.00
-1.00
0.00
1.00
2.00
AXL
EMP3
CAV1
LOXL2
VIM
Zeb2
GATA3
CLDN3
CLDN4
CLDN7
CDH1
Triple Negative Breast Cancer Cells
B
CHARAFE_BREAST_CANCER
LUMINAL_VS_MESENCHYMAL_UP
Enrichment score (ES)
'CL-H' (positively correlated)
C
Relative CDH1 expression
Relative VIM expression
MCF7
BT474
SUM52
BT20
HCC70
HCC38
MDA-231
MDA-157
HBL100
D
RLU (Protease release)
Con
5 μM Erastin
HCC70
HCC38
HBL100
MDA231
HCC70
HBL100
Con
Erastin
(3 d)

Figure S2

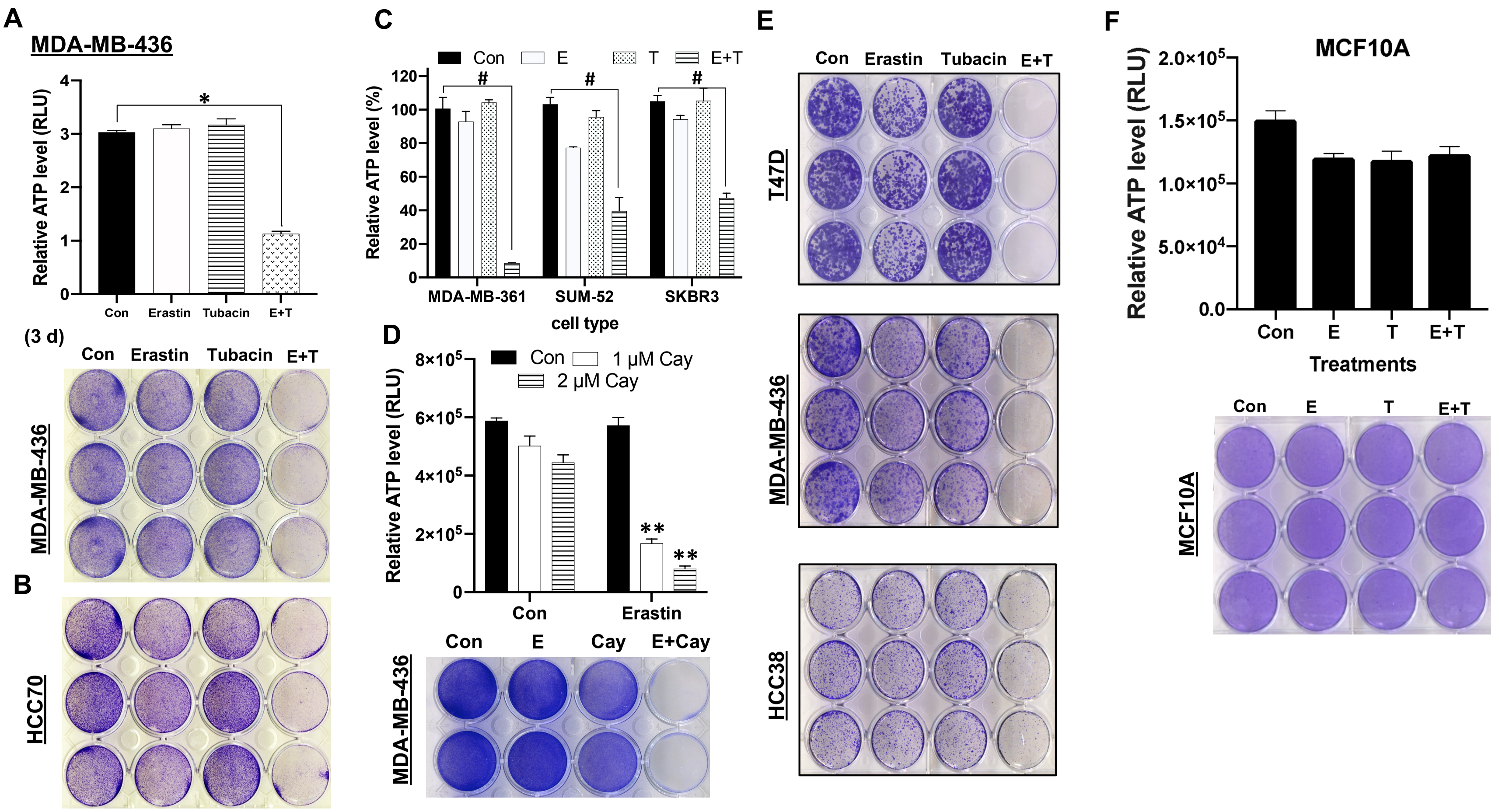
A
MDA-MB-436
Relative ATP level (RLU)
0
1
2
3
4
*
Con
Erastin
Tubacin
E+T
(3 d)
Con
Erastin
Tubacin
E+T
MDA-MB-436
B
HCC70
C
Con
E
T
E+T
Relative ATP level (%)
0
20
40
60
80
100
120
#
MDA-MB-361
SUM-52
SKBR3
cell type
D
Relative ATP level (RLU)
0
2×10^5
4×10^5
6×10^5
8×10^5
Con
1 μM Cay
2 μM Cay
**
Con
Erastin
Con
E
Cay
E+Cay
MDA-MB-436
E
Con
Erastin
Tubacin
E+T
T47D
MDA-MB-436
HCC38
F
MCF10A
Relative ATP level (RLU)
0.0
5.0×10^4
1.0×10^5
1.5×10^5
2.0×10^5
Con
E
T
E+T
Treatments
Con
E
T
E+T
MCF10A

Figure S3

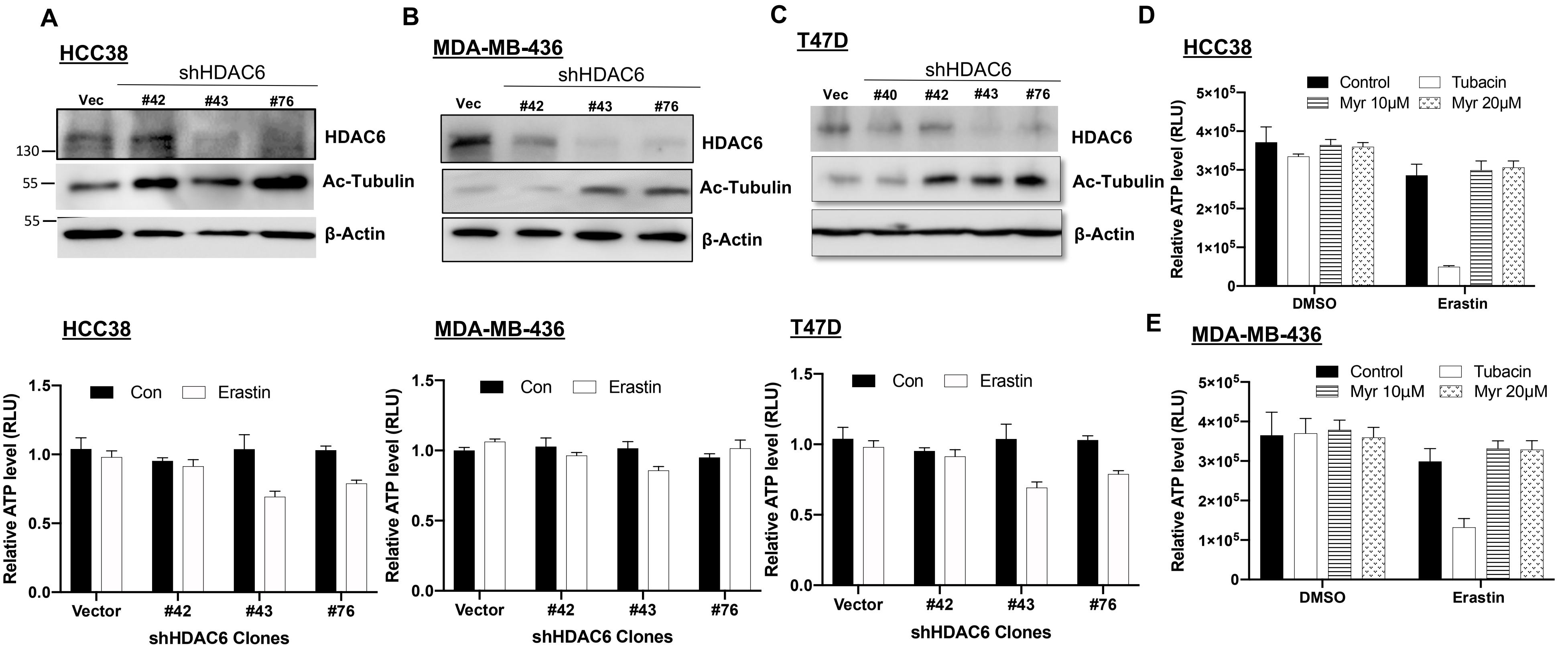
A
HCC38
shHDAC6
Vec
#42
#43
#76
130
55
55
HDAC6
Ac-Tubulin
β-Actin
B
MDA-MB-436
shHDAC6
Vec
#42
#43
#76
HDAC6
Ac-Tubulin
β-Actin
C
T47D
shHDAC6
Vec
#40
#42
#43
#76
HDAC6
Ac-Tubulin
β-Actin
D
HCC38
Control
Tubacin
Myr 10μM
Myr 20μM
Relative ATP level (RLU)
DMSO
Erastin
HCC38
Con
Erastin
Relative ATP level (RLU)
Vector
#42
#43
#76
shHDAC6 Clones
MDA-MB-436
Con
Erastin
Relative ATP level (RLU)
Vector
#42
#43
#76
shHDAC6 Clones
T47D
Con
Erastin
Relative ATP level (RLU)
Vector
#42
#43
#76
shHDAC6 Clones
E
MDA-MB-436
Control
Tubacin
Myr 10μM
Myr 20μM
Relative ATP level (RLU)
DMSO
Erastin

Figure S4

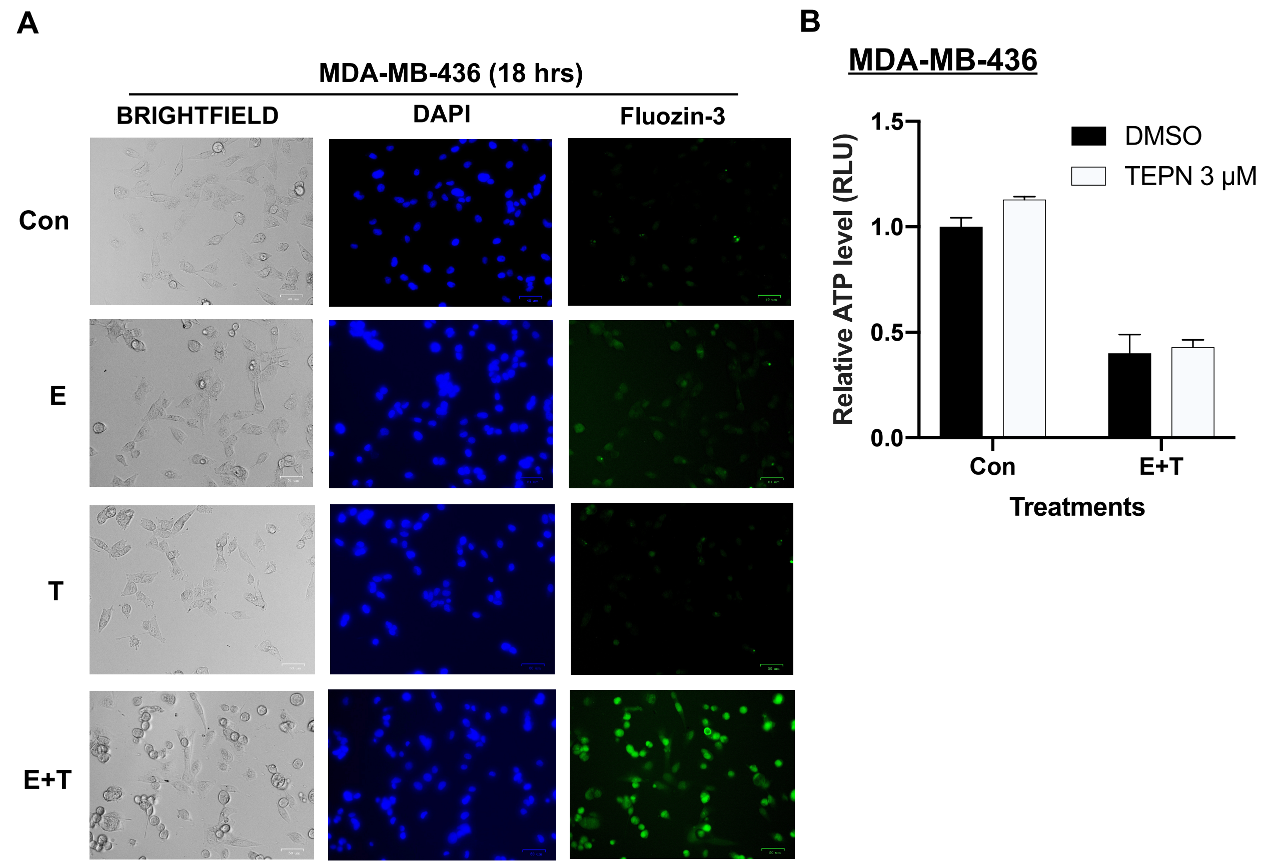
A
MDA-MB-436 (18 hrs)
BRIGHTFIELD
DAPI
Fluozin-3
Con
E
T
E+T
B
MDA-MB-436
Relative ATP level (RLU)
1.5
1.0
0.5
0.0
DMSO
TEPN 3 μM
Con
E+T
Treatments

Figure S5

A

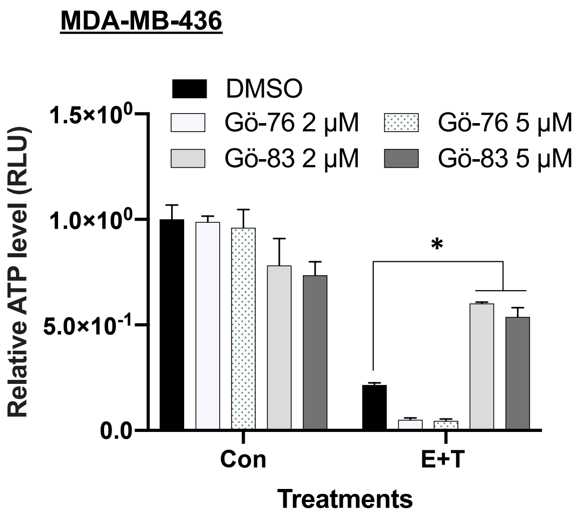

B

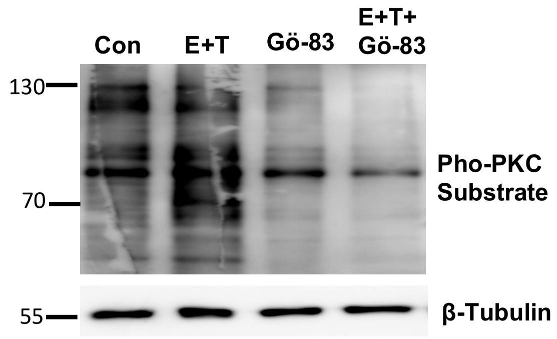

C

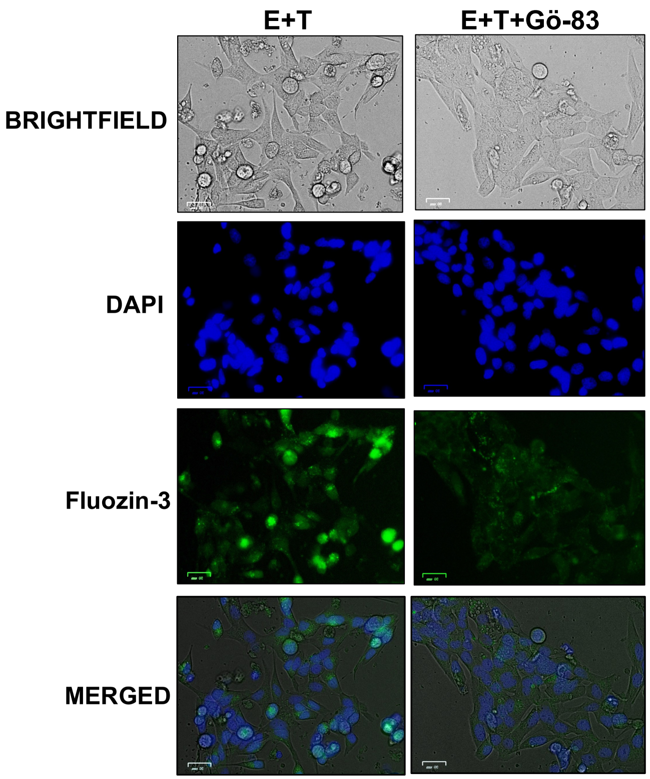

Figure S6

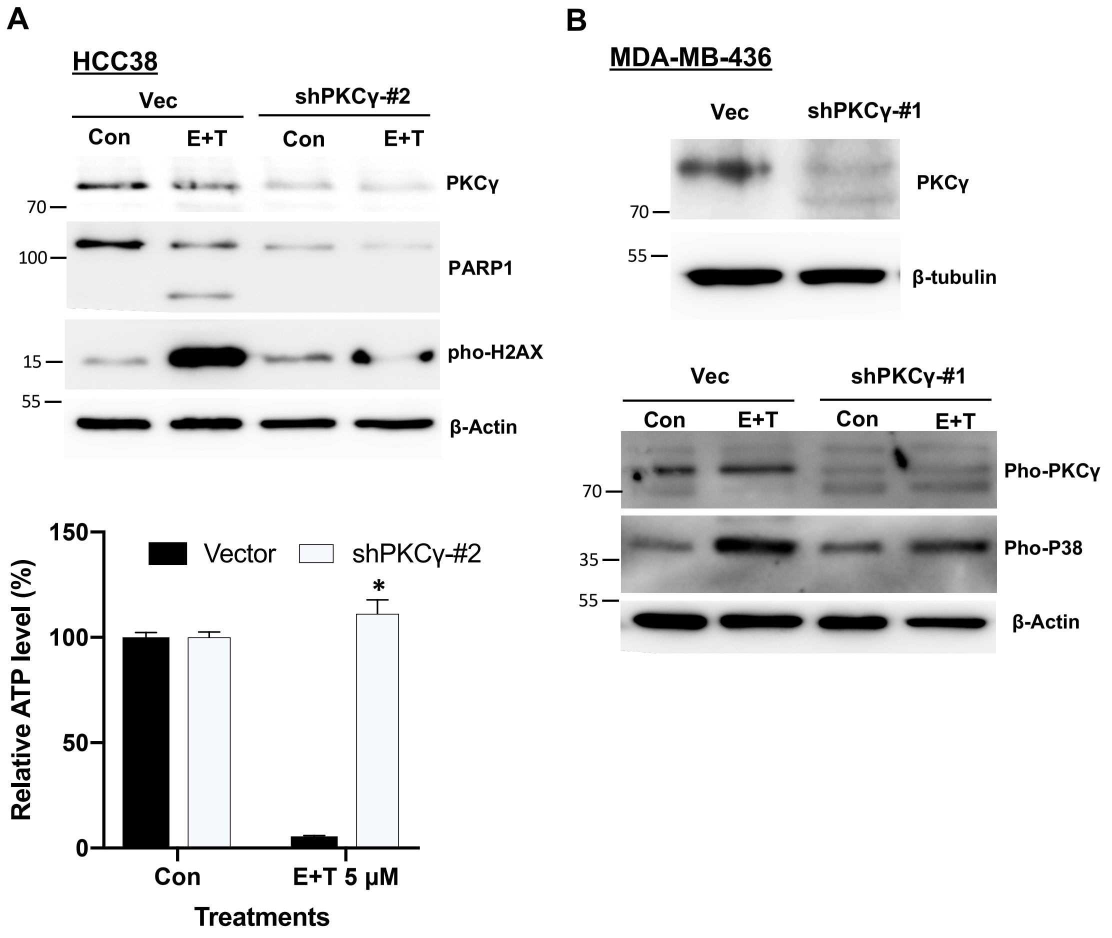
A
HCC38
Vec
shPKCγ-#2
Con
E+T
Con
E+T
PKCγ
70
100
PARP1
15
pho-H2AX
55
β-Actin
Relative ATP level (%)
150
100
50
0
Vector
shPKCγ-#2
*
Con
E+T 5 μM
Treatments
B
MDA-MB-436
Vec
shPKCγ-#1
PKCγ
70
55
β-tubulin
Vec
shPKCγ-#1
Con
E+T
Con
E+T
Pho-PKCγ
70
Pho-P38
35
55
β-Actin

Full blots for Main Figures

Fig 2A

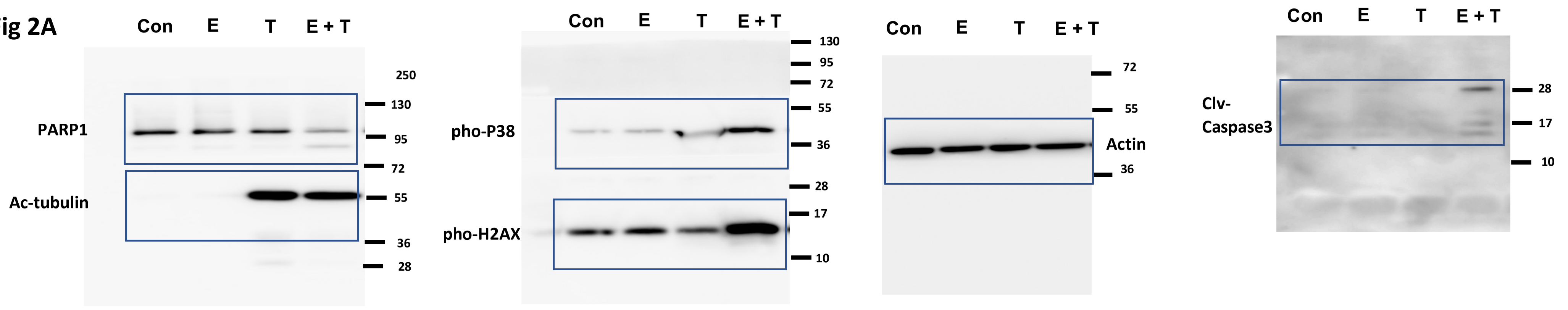

Fig 2B

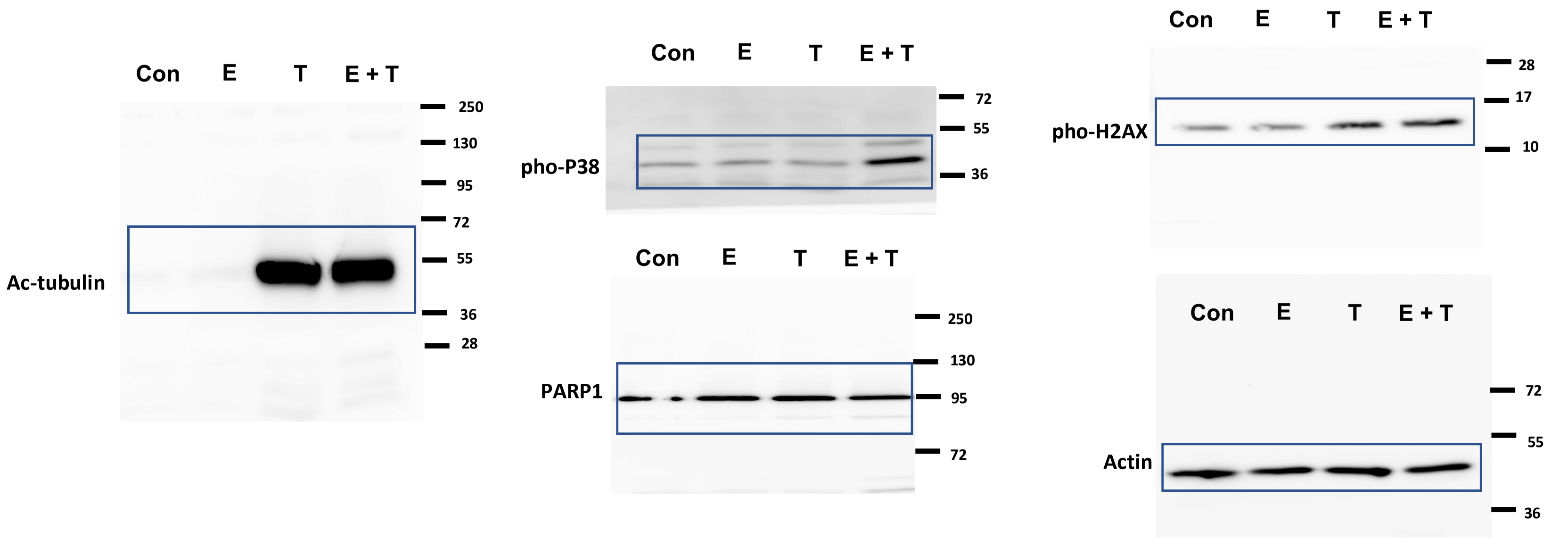

Full blots for Main Figures

Fig 2E

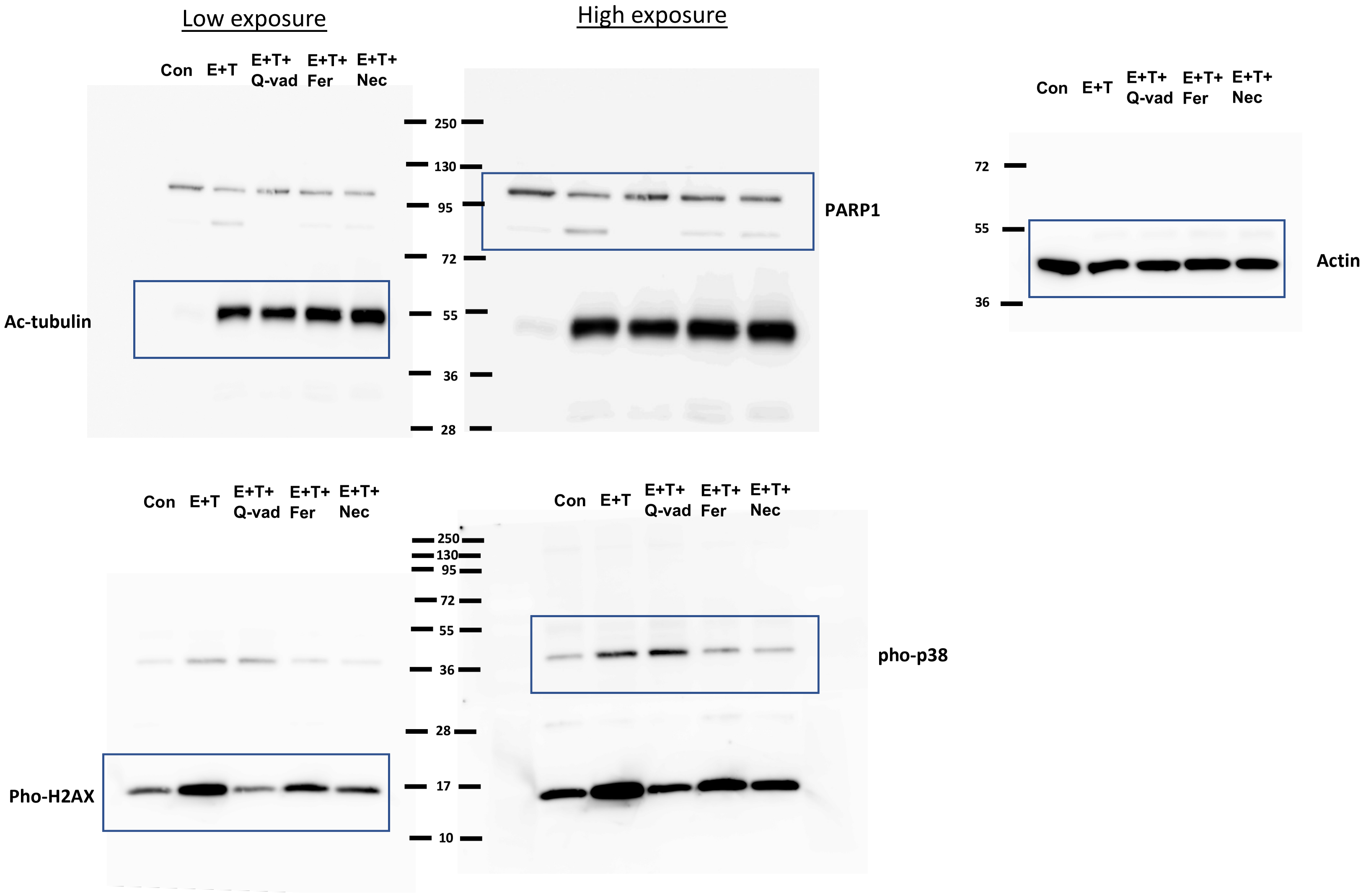

Full blots for Main Figures

Fig 3A

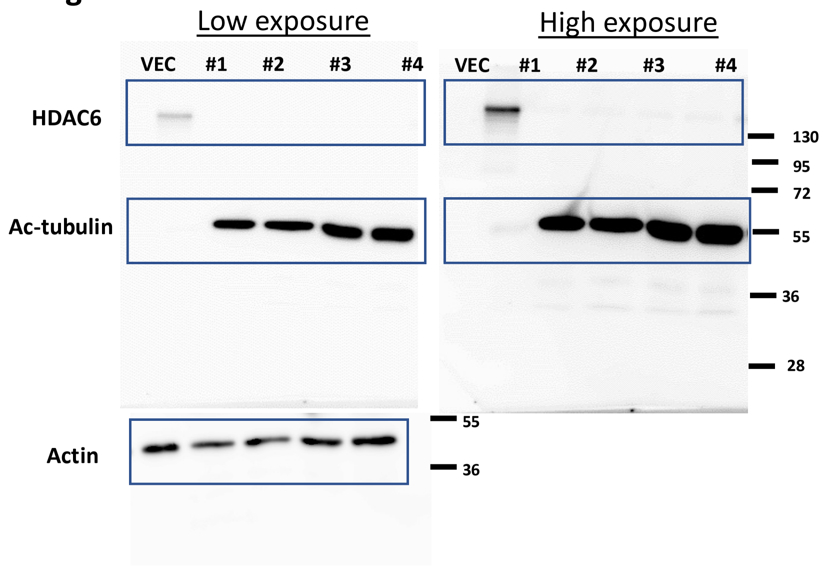

Fig 3B

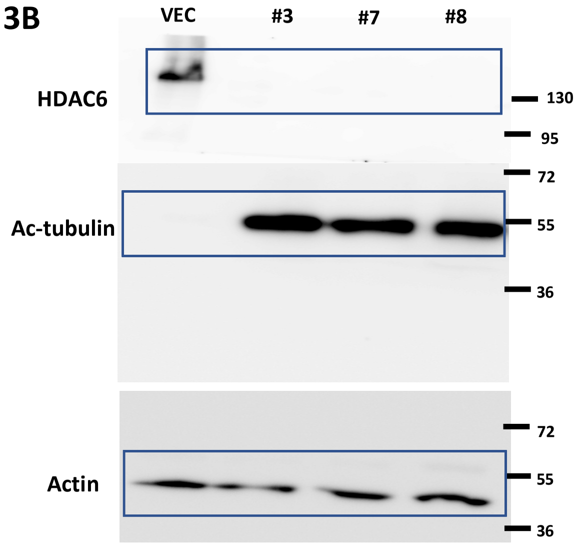

Fig 3C

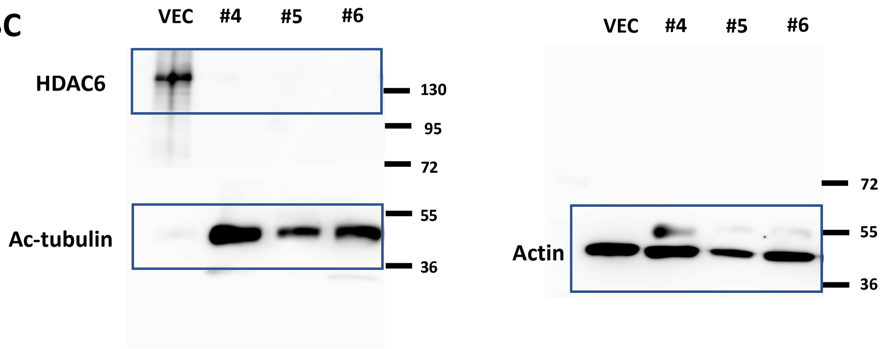

Full blots for Main Figures

Fig 3D

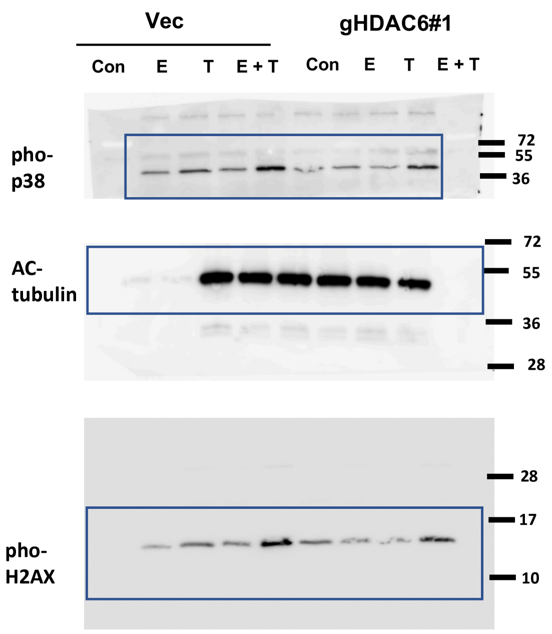

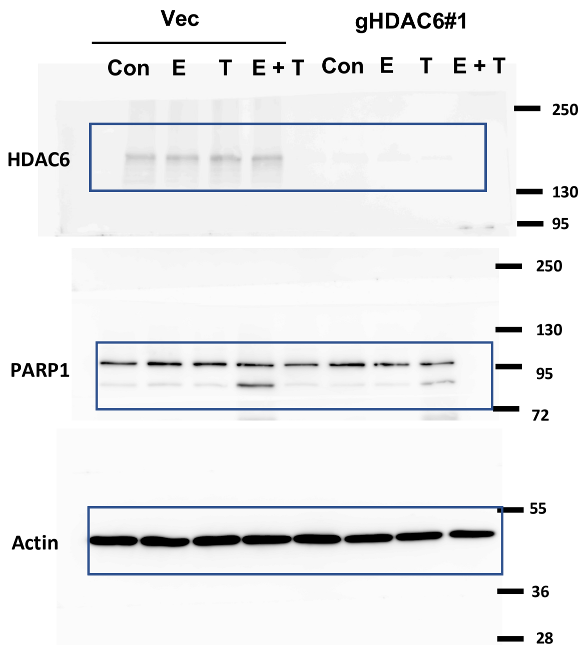

Full blots for Main Figures

Fig 4D

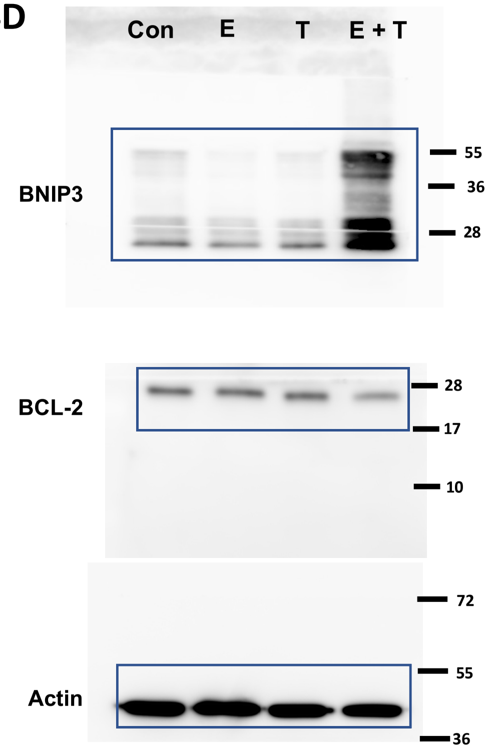

Fig 4F

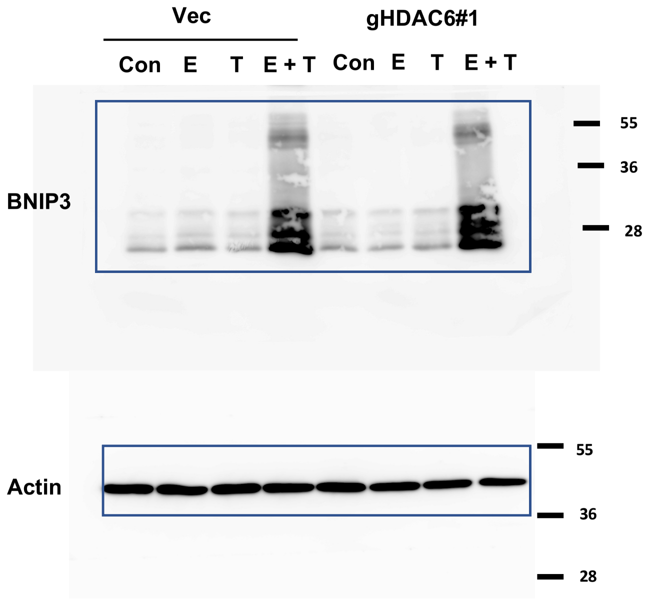

Full blots for Main Figures

Fig 6B

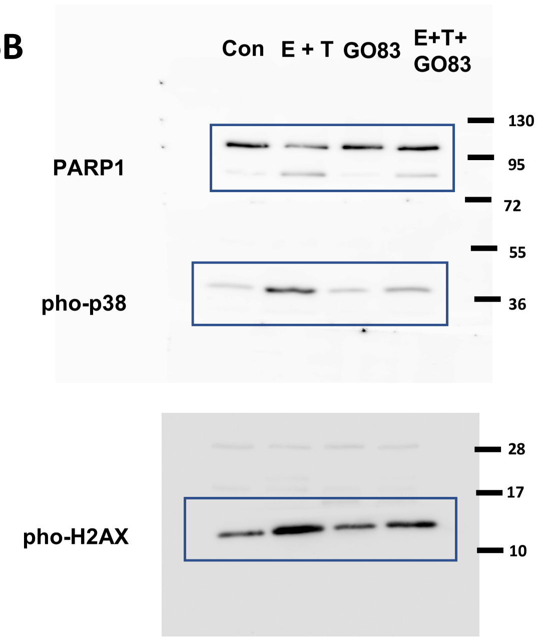

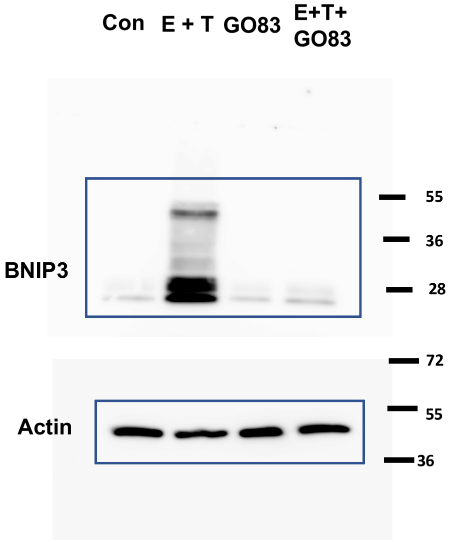

Full blots for Main Figures

Fig 7C

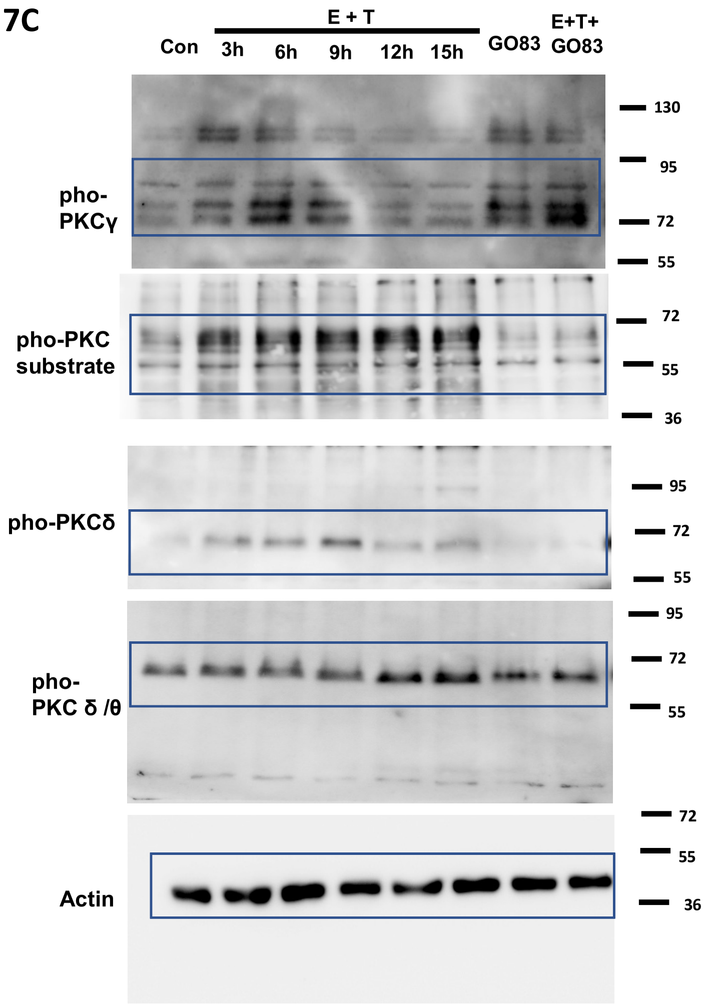

Fig 7E

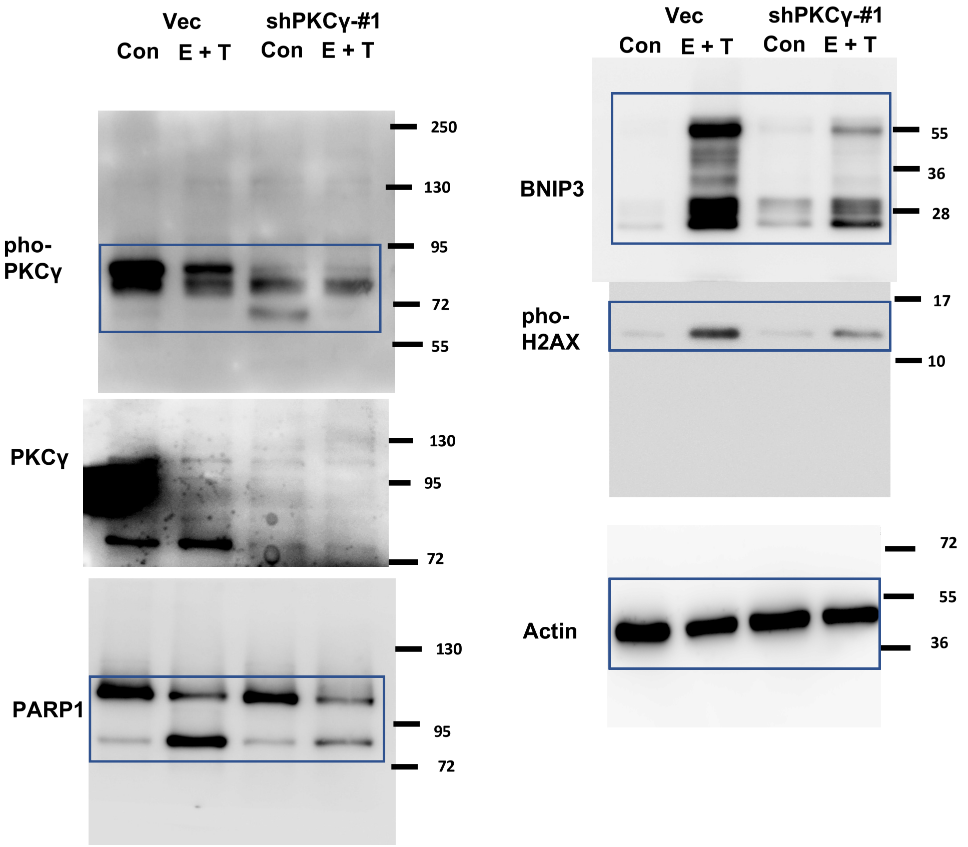

Full blots for Supplemental Figures

Fig S3A

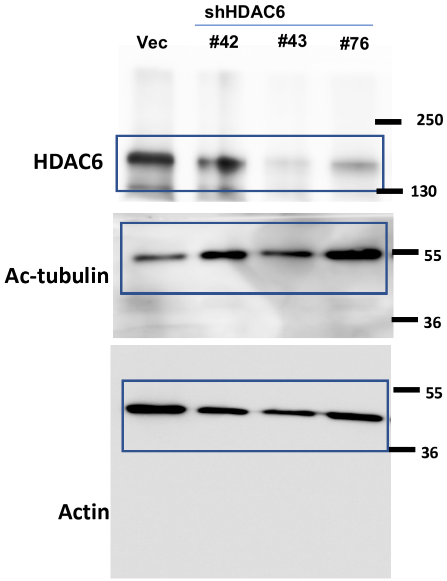

Fig S3B

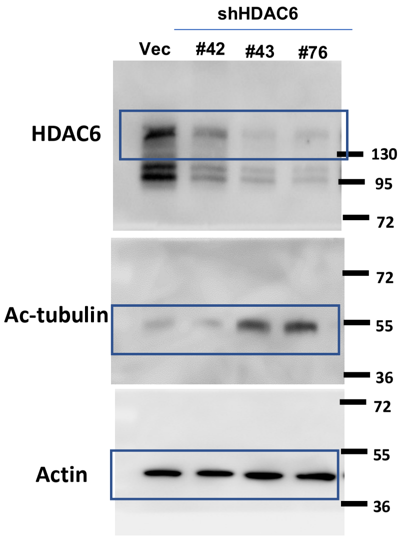

Fig S3C

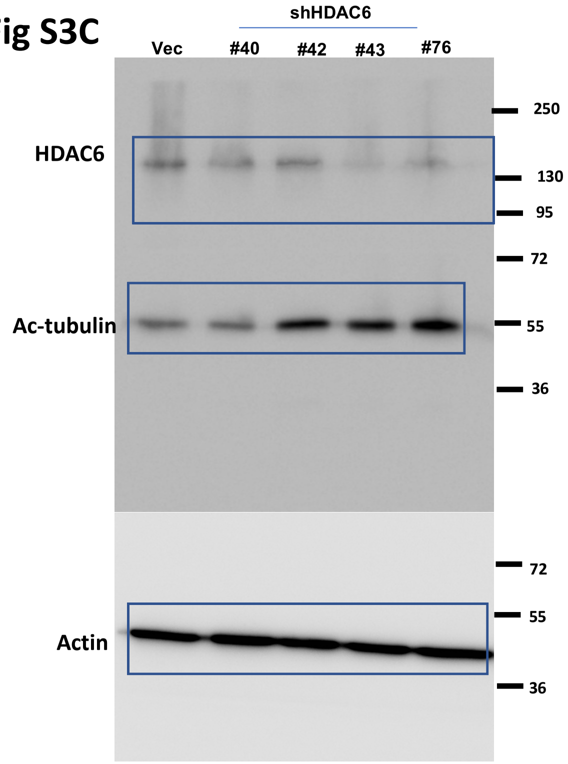

Fig S5B

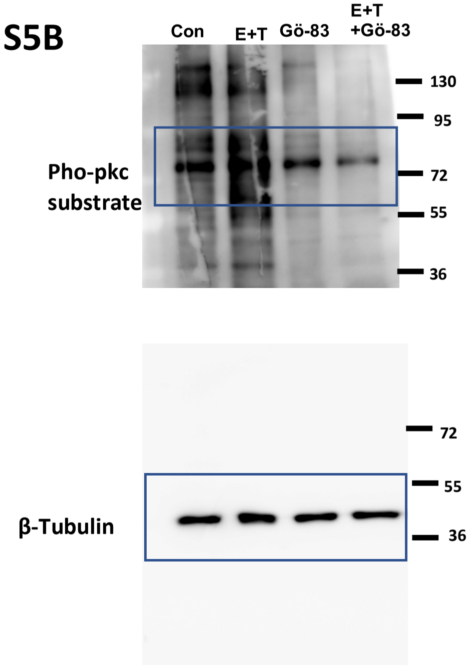

Full blots for Supplemental Figures

Fig S6A

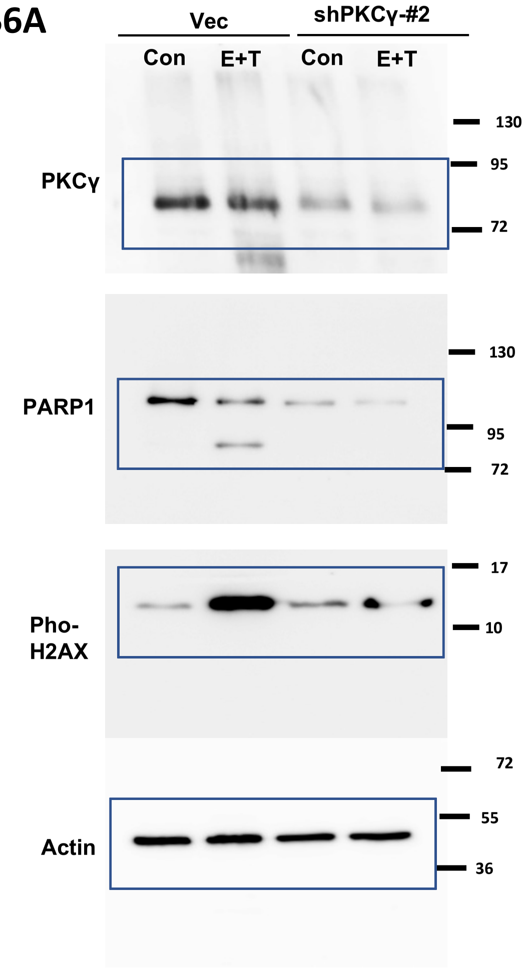

Fig S6B

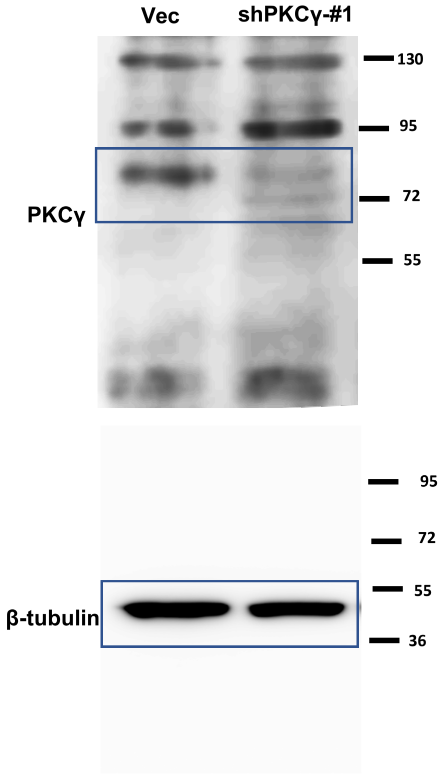

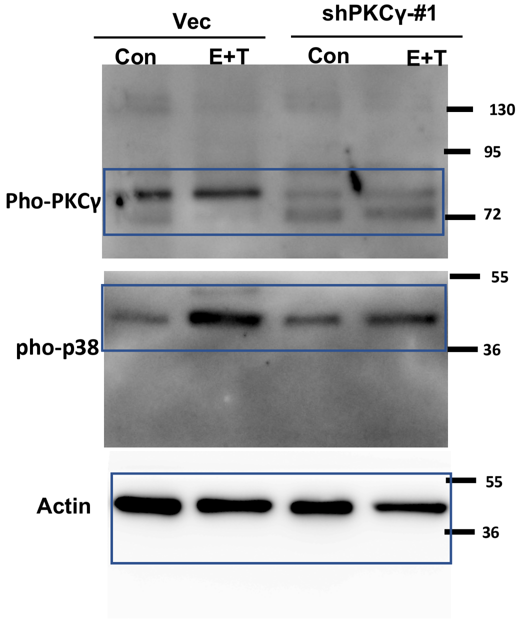

Title: HDAC6 inhibitors sensitize non-mesenchymal triple-negative breast cancer cells to cysteine deprivation

Authors: Tahiyat Alothaim, Morgan Charbonneau, Xiaohu Tang

Supplemental Table 1: Information of all antibodies that were used in this study

Name	Catalog	Company
PARP1	9542S	Cell Signaling Technology
HDAC6	7558T	Cell Signaling Technology
Acetyl-α-Tubulin	5335S	Cell Signaling Technology
phospho-p38	4511S	Cell Signaling Technology
phospho-H2AX	9718S	Cell Signaling Technology
Clv-caspase-3	9661S	Cell Signaling Technology
LC3B	3868S	Cell Signaling Technology
BCL-2	12120S	Cell Signaling Technology
BNIP3	44060S	Cell Signaling Technology
phospho-PKC substrate	2261S	Cell Signaling Technology
Phospho-PKCδ/θ (Ser643/676)	9376T	Cell Signaling Technology
Pan-phospho-PKCγ (Thr514)	9379S	Cell Signaling Technology
PKCγ	59090S	Cell Signaling Technology
Phospho-PKCδ (Thr505)	9374S	Cell Signaling Technology
β-Actin	3700S	Cell Signaling Technology
β-Tubulin	86298S	Cell Signaling Technology

Title: HDAC6 inhibitors sensitize non-mesenchymal triple-negative breast cancer cells to cysteine deprivation

Authors: Tahiyat Alothaim, Morgan Charbonneau, Xiaohu Tang

Supplemental Table 2: Information of all oligoes that were used in this study

qPCR Primer	Oligo sequence
Actin-For	CACTCTTCCAGCCTTCCTTC
Actin-Rev	GGATGTCCACGTCACACTTC
E-Cad-For	CCAGGAGCCAGACACATTTA
E-Cad-Rev	CGGATTAATCTCCAGCCAGT
CLDN3-For	GCCACCAAGGTCGTCTACTC
CLDN3-Rev	CGTAGTCCTTGCGGTCGTAG
CLDN4-For	GGCCTATGGATGAACTGCGT
CLDN4-Rev	AGCCACGATGATGCTGATGA
CLDN7-For	TCTTGCCGCCTTGGTAGCTT
CLDN7-Rev	CCAGGATGACTAGGGCAGAC
Vimentin-For	CCAAACTTTTCCTCCCTGAACC
Vimentin-Rev	GTGATGCTGAGAAGTTTCGTTGA
LOXL2-For	CCCTGGGGAGAGGACATACA
LOXL2-Rev	ATGGAGAATGGCCAGTAGCG
Zeb2-for	CCCTGGCACAACAACGAGAT
Zeb2-rev	GGTCTGGATCGTGGCTTCTG
Bim-For	CATCATCGCGGTATTCGGTT
Bim-Rev	GGTTGCTTTGCCATTTGGTC
BNIP3-For	CCATCTCTGCTGCTCTCTCATT
BNIP3-Rev	TCTTCATCAAAAGGTGCTGGTG
Puma-For	GGAGGGTCCTGTACAATCTC
Puma-Rev	GCTACATGGTGCAGAGAAAG
ZnT1-For	AACACCCTGGTGGCCAATAC
ZnT1-Rev	TCACCACTTCTGGGGTTTTCT
ZnT2-For	TGGGGACAACCTTGACCATC
ZnT2-Rev	GACAGCAGCAGATCACGAAC
MT1G/M/H-For	GGACCCCAACTGCTCCTG
MT1G/M/H-Rev	TCTTCTTGCAGGAGGTGCAT
